# Integrated Transcriptomic and Metabolomics Analysis of the Root Responses of Orchardgrass to Submergence Stress

**DOI:** 10.3390/ijms24032089

**Published:** 2023-01-20

**Authors:** Panpan Shang, Bingna Shen, Bing Zeng, Lei Bi, Minghao Qu, Yuqian Zheng, Yujing Ye, Wenwen Li, Xiaoli Zhou, Xingyun Yang, Yiwei Jiang, Bing Zeng

**Affiliations:** 1 College of Animal Science and Technology, Southwest University, Chongqing 402460, China; 2 Department of Agronomy, Purdue University, West Lafayette, IN 47907, USA

**Keywords:** amino acids biosynthesis, *Dactylis glomerata*, flavonoid biosynthesis, metabolomics, submergence, transcriptomics

## Abstract

Submergence stress can severely affect plant growth. Orchardgrass (*Dactylis glomerata* L.) is an important forage grass, and the molecular mechanisms of orchardgrass to submergence stress are not well understood. The roots of the flood-tolerant cultivar “Dian Bei” were harvested at 0 h, 8 h and 24 h of submergence stress. The combined transcriptomic and metabolomic analyses showed that β-alanine metabolism, flavonoid biosynthesis, and biosynthesis of amino acid pathways were significantly enriched at 8 h and 24 h of submergence stress and were more pronounced at 24 h. Most of the flavonoid biosynthesis-related genes were down-regulated for the synthesis of metabolites such as naringenin, apigenin, naringin, neohesperidin, naringenin chalcone, and liquiritigenin in response to submergence stress. Metabolites such as phenylalanine, tyrosine, and tryptophan were up-regulated under stress. The predominant response of flavonoid and amino acids biosynthesis to submergence stress suggests an important role of these pathways in the submergence tolerance of orchardgrass.

## 1. Introduction

Submergence is a major environmental stress limiting plant growth and development. Plants subjected to complete or partial submergence stress often suffer from oxygen deficiency. It has been found that submergence stress affects plant growth and physiology, including but not limited to reducing shoot and root growth, leaf chlorophyll, carbohydrate content, photosynthetic rate, stomatal conductance, and antioxidant enzyme activities, as well as activating anaerobic fermentation and altering hormone interactions in different plant species [1,2,3,4,5,6,7,8]. The tolerant species or genotypes showed higher root dry weight, chlorophyll content, antioxidant capacity, soluble sugar content and protein levels, and a lesser degree of lipid peroxidation and lower electrolyte leakage in roots or shoots under submergence than the sensitive ones [9,10]. Previous research has indicated some crucial physiological responses associated with plant survival, but the adaptative mechanisms to submergence stress are not fully understood due to genotypic variations in response to stress and recovery from the stress.

At the molecular level, submergence stress largely alters gene expression in plants. Through transcriptomic analyses, differentially expressed genes (DEGs) involved in the key functional and regulatory pathways have been identified in various plant species exposed to submergence stress [11,12,13,14,15,16,17,18]. In bermudagrass (*Cynodon dactylon*), shallow and deep submergence stresses down-regulated the DEGs related to chlorophyll biosynthesis, light harvesting, protein complex, and carbon fixation, while DEGs involved in starch and sucrose hydrolase activities, glycolysis/gluconeogenesis, tricarboxylic acid (TCA) cycle, and oxidative phosphorylation were down-regulated in aboveground tissues but up-regulated in underground tissues [18]. The pathways of glycolysis/gluconeogenesis were also more enriched in submergence-tolerant accessions of *Arabidopsis* [18] and in tolerant soybean (*Glycine max*) [14]. Notably, glycolysis and fermentation genes were strongly induced in both submergence-tolerant *Rorippa sylvestris* and *Rorippa amphibia* and, to a greater extent, in the relatively less tolerant *R. amphibia* [11]. Comparing deepwater rice (*Oryza sativa*) with non-deepwater rice under submergence, DEGs related to gibberellin biosynthesis, trehalose biosynthesis, anaerobic fermentation, cell-wall modification, and ethylene-responsive factors varied significantly in varieties [12]. The results indicate that the activation of certain metabolic pathways, such as glycolysis/gluconeogenesis and fermentation, are associated with plant survival under submergence stress; however, expressions of species-specific responsive genes might contribute to differential underwater growth and adaptation.

Plant response to submergence also involves numerous alterations of metabolites. Metabolomics is one of the emerging and crucial approaches to studying abiotic stress tolerance. Changes in a large scale of metabolites have been identified through metabolomics studies in plants under submergence stress [19,20,21,22]. For example, after soybean plants were exposed to submergence stress, both the primary and secondary metabolism were affected, particularly for those compounds involved in carbon and nitrogen metabolism, as well as the phenylpropanoid pathway [21]. Sugar metabolism was also largely altered in the submergence-tolerant mutant of soybean, and fructose might be the critical metabolite through the regulation of hexokinase and phosphofructokinase to cope with initial submergence stress [20]. When rice was exposed to short-term partial submergence, the metabolite levels in the glycolysis pathway increased in a near-isogenic line with a background of deepwater and non-deepwater rice varieties, while amino acid levels decreased under long-term submergence [23]. Approximately 70% of amino acids detected under submergence increased and then decreased in both tolerant and intolerant wheat (*Triticum aestivum*), but the tolerant cultivar delayed the process of amino acid degradation with an increased time of submergence stress [24]. Willow (*Salix variegate*) is a riparian shrub species that can tolerate long-term complete submergence stress. Metabolites such as ethylene, abscisic acid, jasmonic acid signaling, raffinose family oligosaccharides, highly unsaturated fatty acids, specific stress-related amino acids, organic acid, cell-wall reorganization, and phenylpropanoid metabolic processes (the synthesis of specific phenolics and flavonoids) accumulated and activated in response to 60 d of submergence [25]. In pot mum (*Chrysanthemum morifolium*), the top three types of differentially expressed metabolites were flavone C-glycosides, flavonol, and flavone under submergence stress [26]. Collectively, the results indicate a role of some metabolites in promoting submergence tolerance, but they also suggest complex metabolic responses of plants to submergence stress.

Perennial grasses used for turf and forage are often subjected to periodically submerged conditions in coastal and flood-prone plains. Orchardgrass (*Dactylis glomerata*) is one of the most important forage grasses and is widely distributed in Asia and Europe, as well as in some high-altitude areas in Africa [27,28,29]. This species is also used for grassland improvement and stone desertification management [30,31]. Our previous transcriptomic analysis found that pathways, such as glutathione metabolism, peroxidase, glycolysis, and plant-hormone signal transduction, were highly enriched in the leaves of orchardgrass under submergence stress [32]. Genes involved in metal homeostasis, the antioxidant process, and the secretory pathway were remarkably differentially expressed in leaves between two orchardgrass cultivars under submergence [33]. However, the molecular responses of roots to submergence stress are not known in this species, especially by integrating transcriptomic and metabolomic analyses. Therefore, we designed this experiment to explore transcriptomic and metabolomics alterations in the roots of orchardgrass under submergence stress. The correlations between gene expression and metabolites were analyzed to gain a better understanding of the molecular responses of roots to the stress. The results will be helpful for genetic improvements in orchardgrass aimed at breeding new varieties with enhanced submergence tolerance.

## 2. Results

### 2.1. Root Appearance after Submergence Stress

**Figure 1:**
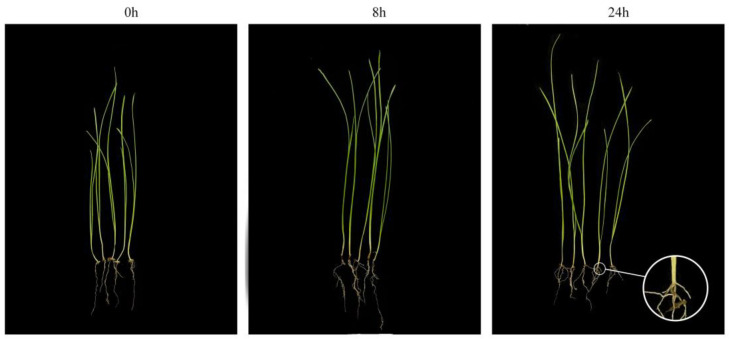
Effect of different duration of submergence stress on the morphology of orchardgrass.

The root tips became darkened after exposure to submergence stress and were more pronounced at 24 h of stress (Figure 1). In addition, adventitious roots were produced at the base of the stem at 24 h of stress, but root length was not significantly altered. Meanwhile, the above-ground parts tended to elongate as the duration of submergence increased. This suggested that short-term submergence stress already induced leaf elongation of the orchardgrass, which might help resist the extreme hypoxic environment.

### 2.2. Transcriptome Sequencing Analysis and Identification of DEGs

The raw data were filtered, checked for sequencing error rates and GC content distribution, and then clean reads (Table S1) were obtained for subsequent analysis. The clean reads were mapped to the orchardgrass reference genome using HISAT2 software (Table S2). The results showed that the proportion of sequenced reads successfully mapped to the genome was higher than 70%, indicating the high reliability of the sequencing data. The correlation of gene expression levels between samples showed good intra-group reproducibility (Figure S1). 

**Figure 2:**
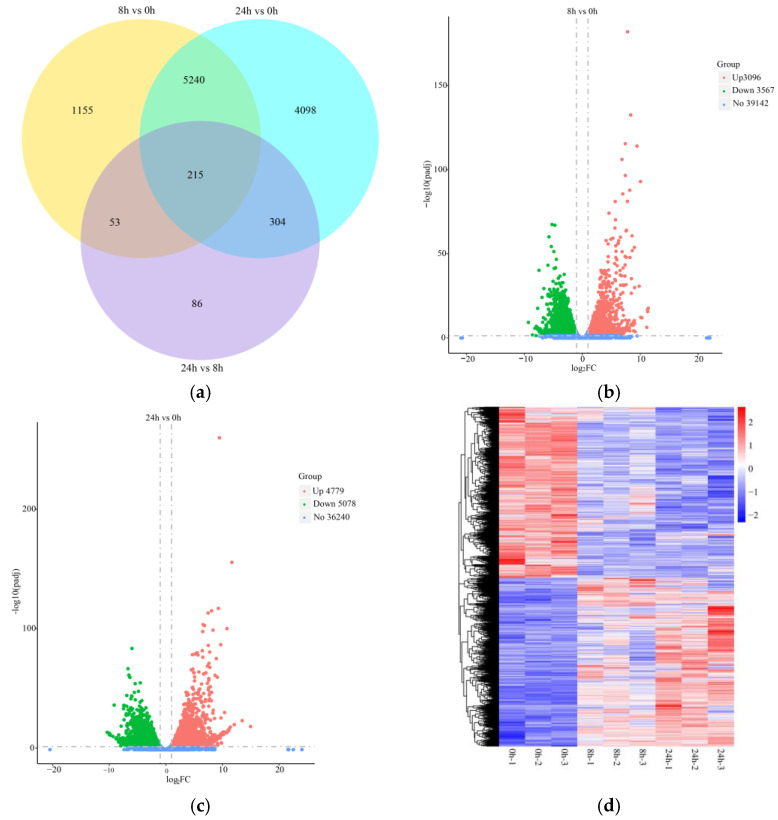
Statistical analysis of the number of differentially expressed genes (DEGs) and expression pattern in the roots of orchardgrass under submergence stress. (**a**) Venn diagram of the number of DEGs. Volcanic map of up- and down-regulated genes for 8 h vs. 0 h (**b**) and 24 h vs. 0 h (**c**). (**d**) Cluster heat map of DEGs was shared in all treatment groups.

Based on the sequencing data, we analyzed the RNA-Seq at 0 h, 8 h, and 24 h to reveal the DEGs under submergence stress. The distribution of DEGs in different treatment groups is shown in Figure 2a. A total of 6663 DEGs (3096 up-regulated and 3567 down-regulated) were identified in the roots after 8 h of submergence, while 9857 DEGs (4779 up-regulated and 5078 down-regulated) were identified at 24 h of treatment (Figure 2b,c). Among them, 5455 DEGs were co-expressed. The gene expression pattern changed significantly after submergence stress (Figure 2d).

### 2.3. GO Analysis and KEGG Pathway Analysis

**Figure 3:**
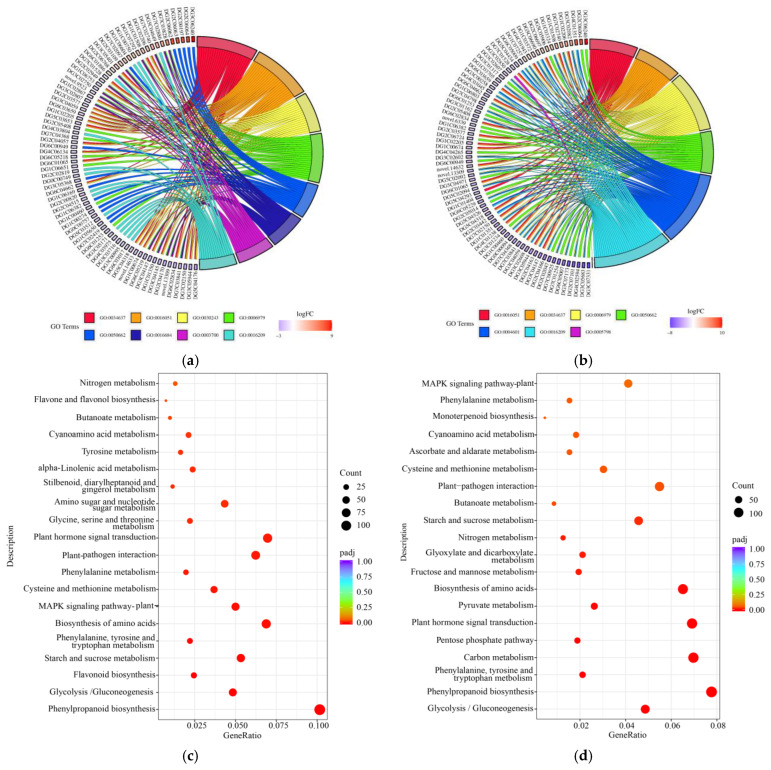
Functional enrichment analysis of differentially expressed genes (DEGs) in the roots of orchardgrass under submergence stress. 8 h vs. 0 h (**a**) and 24 h vs. 0 h (**b**). Partial GO terms chord diagram. Bubble diagram of top 20 enrichment pathways for 8 h vs. 0 h (**c**) and 24 h vs. 0 h (**d**).

To fully understand the functional properties of DEGs and the metabolic pathways of gene products, we performed functional enrichment analyses. Each group of DEGs was annotated into three categories: biological process; molecular function; and cellular component (Figure S2). The Gene Ontology (GO) enrichment analysis revealed that most of the terms had more down-regulated DEGs than up-regulated DEGs and more DEGs at 24 h than at 8 h. The DEGs were highly expressed in terms of oxidative stress, coenzyme-binding oxidoreductase activity, acting on peroxide as an acceptor, antioxidant activity, etc., under stress. Unlike at 8 h, the genes related to peroxidase activity were more active at 24 h. Moreover, some highly expressed genes were involved in multiple regulatory pathways simultaneously under 8 h and 24 h of submergence stress (Figure 3a,b, Table S3). 

The Kyoto Encyclopedia of Genes and Genomes (KEGG) enrichment was analyzed to show the effect of submergence stress on the metabolic pathways of DEGs. The top ranked 20 metabolic pathways were significantly enriched at 8 h and 24 h, compared to 0 h, including phenylpropanoid biosynthesis, plant-hormone signal transduction, the biosynthesis of amino acids, starch and sucrose metabolism, plant–pathogen interaction, and MAPK signaling pathway–plant (Figure 3c,d). The number of DEGs in each metabolic pathway was higher at 24 h than at 8 h (Table S4). The antioxidant-related metabolic pathways, such as ascorbate and aldarate metabolism and glyoxylate and dicarboxylate metabolism, were significantly enriched at 24 h of submergence.

### 2.4. qRT-PCR Validation

To verify the reliability of the transcriptome sequencing data, we performed qRT-PCR validation on the 11 screened DEGs. Correlation analysis of qPCR and RNA-Seq results for these 11 genes revealed that R^2^ was 0.931 at 8 h and 0.806 at 24 h of submergence stress. The validation results were basically consistent with the expression level of RNA-seq sequencing results (Figure S3). This indicated that the data obtained by transcriptome sequencing were reliable.

### 2.5. Quality Control of Metabolomic Data

**Figure 4:**
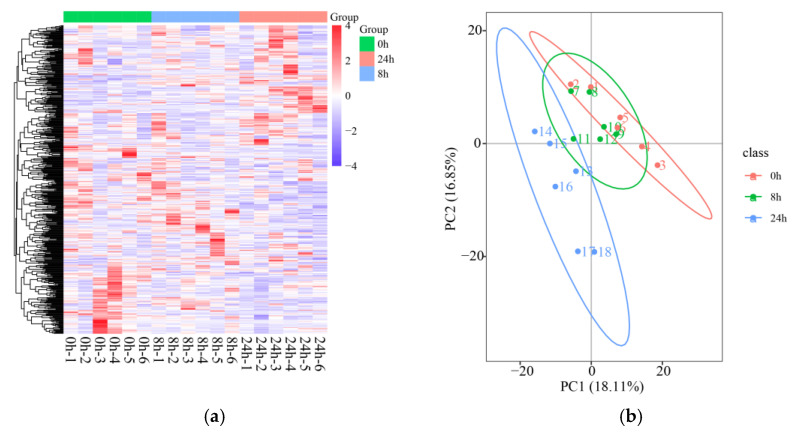
Quality control chart of metabolome data in the roots of orchardgrass under submergence stress. (**a**) Total metabolite hierarchical clustering heat map. (**b**) PCA analysis of the expression of metabolites for 0 h, 8 h, and 24 h.

A total of 439 metabolites were obtained from a control group of root samples extracted from 0 h of submergence stress and 8 h and 24 h as treatment groups for untargeted metabolomic analyses. A hierarchical clustering heat map was constructed and annotated with Human Metabolome Database (HMDB) classification. The phenylpropanoid- and polyketide-related metabolites had the highest number under submergence stress (Figure 4a and Figure S4). Correlation analysis of the QC samples showed R^2^ values all close to 1 (Figure S5), indicating that the entire analysis process was stable and reproducible. Subsequently, principal component analysis (PCA) was performed, and there was a significant separation between 0 h, 8 h, and 24 h (Figure 4b). PC1 and PC2 explained more than 35.0% of the variability, and metabolites were mainly distinguished by PC1 (Figure 4b). Through partial least-squares discrimination (PLS-DA) analysis, treatment groups were separate from each other, with both R^2^ and Q^2^ close to 1 (Figure S6), indicating a stable and reliable relationship between metabolite expression and sample categories.

### 2.6. Differentially Expressed Metabolites (DEMs) and Functional Enrichment 

**Figure 5:**
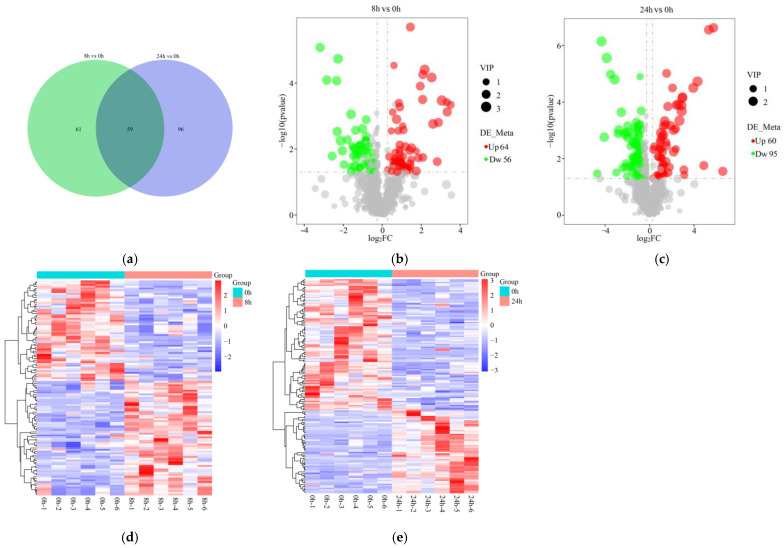
Statistical analysis of differentially expressed metabolites (DEMs) in the roots of orchardgrass under submergence stress. (**a**) Venn diagram of the number of DEMs. Volcanic map of up- and down-regulated DEMs in roots of orchardgrass at 8 h vs. 0 h (**b**) and 24 h vs. 0 h (**c**). Heat map of DEMs in root at 8 h vs. 0 h (**d**) and 24 h vs. 0 h (**e**).

A total of 120 DEMs were obtained at 8 h of submergence compared to 0 h (Figure 5a). Of them, 64 were up-regulated and 56 were down-regulated (Figure 5b). A total of 155 DEMs were obtained at 24 h, compared to 0 h, including 60 up-regulated and 95 down-regulated (Figure 5a,c). Subsequently, the hierarchical clustering analysis showed that DEMs were clustered at different levels at different time points (Figure 5d,e).

**Figure 6:**
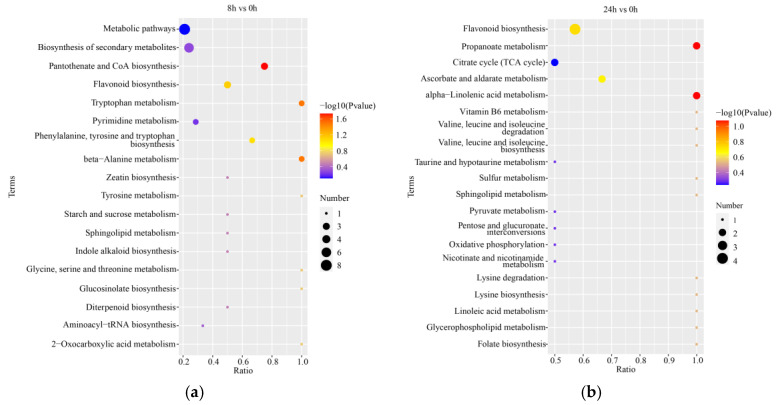
Functional enrichment and correlation analysis of differentially expressed metabolites (DEMs) in the roots of orchardgrass under submergence stress. Bubble diagram of T20 enrichment pathway in 8 h vs. 0 h (**a**) and 24 h vs. 0 h (**b**).

The DEMs were subjected to KEGG-enrichment analysis, and the top 20 enriched pathways were identified (Table S5). The top five enriched metabolic pathways after 8 h of stress were pantothenate and CoA biosynthesis, tryptophan metabolism, β-alanine metabolism, flavonoid biosynthesis, and phenylalanine, tyrosine and tryptophan biosynthesis; meanwhile, the zeatin biosynthesis pathway was also significantly enriched at 8 h (Figure 6a). The top five metabolic pathways enriched after 24 h of treatment were propanoate metabolism, alpha-linolenic acid metabolism, ascorbate and aldehyde metabolism, flavonoid biosynthesis, and vitamin B6 metabolism (Figure 6b). Flavonoid biosynthesis was significantly enriched in the roots at both 8 h and 24 h of stress. 

Subsequently, the DEMs with *p*-value < 0.05 and ranked in the top 20 (from smallest to largest *p*-values) were screened for correlation analyses and categorical annotation to verify synergistic or mutually exclusive relationships between different metabolites (Figure S7). Flavonoid biosynthesis-related metabolites naringenin chalcone and naringenin were significantly enriched at 8 h of submergence stress, while naringenin chalcone, naringenin, apigenin, and scutellarin in this class were enriched at 24 h of treatment (Table S6). All the compounds in this class showed extremely strong synergistic effects. Moreover, amino acid biosynthesis-related metabolites, such as tryptophan, gamma-aminobutyric acid, and n-epsilon-acetyllysine, were also significantly enriched and showed synergistic relationships with each other (Table S6). However, at 8 and 24 h of stress, the metabolite jasmonic acid had mutually exclusive relationships with most of the metabolites except for dihydrojasmone and n-[(−)-Jasmonoyl]-(l)-isoleucine, which showed strong synergistic relationships with each other (Table S6).

### 2.7. C orrelation and Enrichment Analysis between DEGs and DEMs 

**Figure 7:**
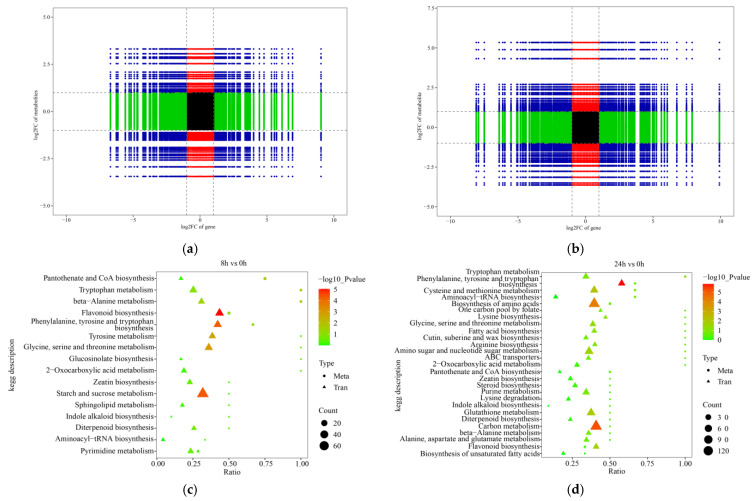
Correlation analysis and functional enrichment of differentially expressed genes (DEGs) and differentially expressed metabolites (DEMs). Nine-quadrant map of genes and metabolites in roots at 8 h vs. 0 h (**a**) and 24 h vs. 0 h (**b**). Association analysis of KEGG pathway with DEGs and DEMs in roots at 8 h vs. 0 h (**c**) and at 24 h vs. 0 h (**d**).

To further analyze the role of genes and metabolites in the regulation of orchardgrass under submergence stress, a nine-quadrant diagram was drawn to illustrate the correlation between genes and metabolites. Among them, metabolite abundance located in quadrants one, two, and four was higher than gene abundance, indicating that metabolites were up-regulated while genes remained unchanged or down-regulated. Metabolite abundance located in the sixth, eighth, and ninth quadrants was lower than gene abundance, indicating that genes were up-regulated but metabolites remained unchanged or down-regulated. Only genes and metabolites located in the third and seventh quadrants showed consistent differential expression patterns between DEGs and DEMs. A total of 234 metabolites were positively regulated by 2389 genes at 8 h of submergence, and a total of 286 metabolites were positively regulated by 2389 genes at 24 h of stress (Figure 7a,b). These results suggest that these changes in metabolite accumulation might be directly or indirectly regulated by the corresponding genes.

The DEGs and DEMs were simultaneously subjected to KEGG pathway analysis. The results showed that 16 and 27 metabolic pathways were enriched at 8 h vs. 0 h and at 24 h vs. 0 h, respectively. Among them, two metabolites and 17 genes were significantly enriched to β-alanine metabolism, three metabolites and 30 genes to flavonoid biosynthesis, and two metabolites and 27 genes to phenylalanine, tyrosine, and tryptophan biosynthesis at 8 h of submergence stress (Figure 7c). Three metabolites and 114 genes were enriched for the biosynthesis of amino acids, two metabolites and 29 genes for the biosynthesis of flavonoids, two metabolites and 53 genes for the metabolism of cysteine and methionine, and two metabolites and 37 genes for the biosynthesis of phenylalanine, tyrosine, and tryptophan biosynthesis at 24 h of submergence (Figure 7d). In addition, the metabolic pathways of zeatin biosynthesis, pantothenate, and CoA biosynthesis were significantly enriched at 8 h and 24 h of stress.

### 2.8. Biosynthesis of Flavonoid and Amino Acids

**Figure 8:**
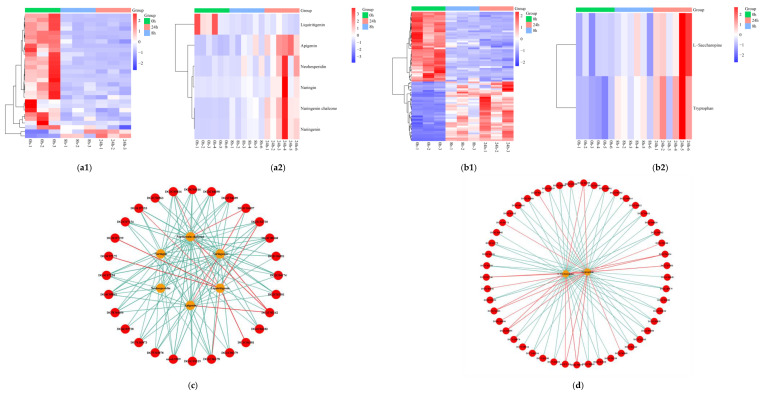
Analysis of differentially expressed genes (DEGs) and differentially expressed metabolites (DEMs) involved in biosynthesis of flavonoid and amino acids in the roots of orchardgrass under submergence stress. Expression pattern of DEGs and DEMs involved in flavonoid biosynthesis (**a1**,**a2**) and amino acids (**b1**,**b2**). Correlation network of DEGs and DEMs involved in flavonoid biosynthesis (**c**) and amino acids (**d**). Red circles indicate genes and yellow circles indicate metabolites. Lines colored in “red” and “green” represent positive and negative correlations, respectively.

Because the flavonoid biosynthesis and biosynthesis of amino acids pathways were significantly enriched through the integrated DEGs and DEMs analysis under submergence, the expression patterns and network interactions of the DEGs and DEMs annotated in these two pathways were analyzed. Most DEGs associated with flavonoid biosynthesis were down-regulated in expression after submergence stress, while the metabolites naringenin, apigenin, naringin, neohesperidin, and naringenin chalcone, were up-regulated in expression, suggesting that orchardgrass may regulate the biosynthesis of flavonoid-related substances by down-regulating related genes in response to submergence stress (Figure 8a1,a2). The genes involved in the biosynthesis of the amino acid pathway were more significantly up-regulated at 24 h of submergence, and the metabolites tryptophan and l-saccharopine were both up-regulated at 24 h of stress (Figure 8b1,b2). The network analysis of DEGs and DEMs annotated in these two metabolic pathways showed positive and negative regulations of genes and metabolites (Table S7a,b; Figure 8c,d).

## 3. Discussion

Through the combined transcriptomic and metabolomics analyses, the post-transcriptional state of related gene expression can be explored to further understand the response mechanisms of orchardgrass to submergence stress. When the roots of orchardgrass were exposed to 8 h and 24 h submergence, genes involved in flavonoid biosynthesis and amino acid biosynthesis were significantly expressed, and the content of compounds, such as flavonoids and amino acids, also showed an increasing trend. The results indicate that the flavonoid and amino acid biosynthetic pathways largely responded to submergence stress in orchardgrass. Similar results were found in the aboveground tissue of red clover (*Trifolium pratense*) to Pb toxicity [34,35].

Flavonoids participate in plants’ responses to environmental stress and serve as strong antioxidants and free radical scavengers [36]. A study on the radical scavenging and antioxidant activities of flavonoids from *Scutellaria baicalensis* extracts concluded that the scavenging activities of different flavonoids varied in response to different environmental conditions [37]. Submergence stress increased lipid peroxidation of roots and decreased antioxidant activities in four genotypes of perennial ryegrass under submergence stress [4]. In this study, naringenin, apigenin, naringin, neohesperidin, and naringenin chalcone involved in flavonoid biosynthesis were significantly enriched at 24 h of submergence stress. Flavonoid compound accumulation was also associated with the submergence tolerance of willow [25] and pot mum [22]. The results suggest the role of flavonoid in coping with oxidative injury from submergence stress.

It has been shown that the flavonoid biosynthetic pathway is regulated by different classes of transcription factors (TFs), such as MYB, WRKY, NAC and bHLH [38,39,40]. Among these TFs, the MYB-bHLH-WD40 ternary complex is one of the most important regulatory components involved in the biosynthesis of phenylpropanoids and flavonoids [22,41,42]. In this study, the metabolic pathway of flavonoid biosynthesis was enriched at 8 h and 24 h of submergence stress, accompanied by a high expression of genes encoding the TF family, such as WRKY and MYB. The results indicate that these TFs could regulate the biosynthesis of flavonoid compounds in orchardgrass roots under the water.

Amino acids are important physiologically active organic compounds in plants and play an important role in regulating growth. When plants experience abiotic stress, free amino acids can respond rapidly by increasing the concentration of certain amino acids for adaptation to environmental changes [43,44]. In this study, biosynthesis of amino acids and β-alanine metabolism were enriched at both the gene and metabolite levels in the roots of orchardgrass in response to submergence. Similar to the results of orchardgrass root under submergence stress, alpha-linolenic acid metabolism, phenylalanine metabolism, and phenylpropanoid biosynthesis pathways were also significantly enriched in the leaf transcriptome analysis [32]. Meanwhile, oxidoreductase activity, cofactor bling, and oxidation–reduction processes are highly present in GO-enriched terms, suggesting that the leaves of orchardgrass have a complex oxidoreductase process in response to flooding stress. However, carbon metabolism, flavonoid biosynthesis, and zeatin biosynthesis pathways were not significantly enriched in the leaves [32]. The up-regulated expression of genes encoding β-alanine metabolism, methionine metabolism, tyrosine metabolism, glycine, serine, and threonine metabolism was also found in field cress (*Sesbania cannabina*) roots after 3 h of submergence stress [45]. In wheat, most of the measured amino acids increased in shoots during the first 12 days of submergence, with only five showing decreasing or unchanged levels, including alanine in both tolerant and sensitive cultivars. However, all amino acid levels were lower in the intolerant cultivar than in the tolerant cultivar on days 14 and 16 of treatment [24]. All results suggest that amino acid metabolism-related processes, such as β-alanine metabolism and phenylalanine metabolism, are associated with submergence tolerance, but the responses of amino acids to submergence varies with plant species, stress duration and intensity. We speculated that the increased amino acids in the roots of orchardgrass after submergence stress was probably due to accelerated nitrogen metabolism to make the raw material for more abundant protein biosynthesis.

The metabolic pathways of zeatin biosynthesis, pantothenate, and CoA biosynthesis were significantly enriched in orchardgrass at 8 h and 24 h of submergence stress, suggesting a role of these pathways in submergence tolerance. Zeatin is a natural cytokinin present in plants, which promotes plant-cell division, prevents chlorophyll and protein degradation, maintains cell viability, and delays plant aging [46,47]. The up-regulated zeatin biosynthesis found in this study indicated its role in promoting growth and assisting submergence tolerance in orchardgrass. The enriched DEGs for zeatin biosynthesis were also found in *Populus ussuriensis* exposed to salt stress [48]. CoA is an essential cofactor in the metabolism and is particularly important in the metabolism of fatty acids. A combined transcriptomic and metabolomic analysis of the response of *Zygophyllum* plants to salt stress revealed that pantothenate and CoA biosynthesis were significantly enriched in both salt-tolerant and sensitive varieties [49], demonstrating its role in altering plant response to stress conditions.

## 4. Materials and Methods

### 4.1. Plant Materials, Submergence Treatment and Harvesting

The flood-tolerant orchardgrass variety ‘Dian Bei’ was used for this experiment. The seeds were obtained from the College of Grassland Science and Technology, Sichuan Agricultural University, Chengdu, China. The seeds were placed in Petri dishes containing two layers of moist filter paper and germinated in dark conditions in an incubator at 22 °C. After germination, the uniformly growing plants were transplanted into pots (15.0 cm in diameter and 13.5 cm in height), containing vermiculite, perlite, and nutrient soil (1:1:3, *v*/*v*/*v*), and placed in an incubator with temperatures of 22/15 °C (day/night), 70–85% humidity, and an 8 h photoperiod of 100 µmol·m^−2^·s^−1^. Submergence stress treatment started when the plants grew 3–4 leaves. Plants with the same overall growth were selected and placed in a water tank (length 80 cm × width 57 cm × height 50 cm), and water was added to completely submerge the plants in water. The intact roots were harvested at 0 h, 8 h, and 24 h after submergence stress, with five biological replicates at each time point. The morphological changes were observed immediately.

### 4.2. Transcriptome Sequencing and Data Analysis

At 0 h, 8 h, and 24 h of submergence stress, approximately 5 g of the root system was taken for each sample, rapidly placed in liquid nitrogen, and then stored in a freezer at −80 °C. Three biological replicates were made at each time point. Total RNA was extracted using a Trizol kit (Invitrogen, Carlsbad, CA, USA), and the samples were tested for RNA integrity and purity by agarose gel electrophoresis using a Nano Photometer spectrophotometer (Implen, Westlake Village, CA, USA) and an Agilent 2100 bioanalyzer (Agilent Technologies, Santa Clara, CA, USA). The samples that met the requirements were sequenced by Illumina PE150 (Illumina, San Diego CA, USA) at Beijing NovoMagic.

The sequencing raw data were filtered by removing low-quality reads and detecting sequencing error rate and GC content distribution. The clean reads were mapped to the orchardgrass reference genome [50,51] using HISAT2 software (http://ccb.jhu.edu/software/hisat2/faq.shtml, accessed on 20 July 2021) to obtain gene-on-gene localization information, as well as information on sequence characteristics specific to this species. Gene alignment statistics and fragments per kilobase of exon per million mapped fragments (FPKM) values were calculated. Differential expression results were analyzed for each comparison of treatment time points using DESeq2 (version 1.16.1) software [52], and padj < 0.05 and |log2FC| ≥ 1 were used as screening criteria for identifying the DEGs. To further implement the functions of DEGs, we used cluster profiler software to perform GO functional enrichment and KEGG-pathway-enrichment analysis.

### 4.3. qRT-PCR Analysis

Eleven DEGs with expression in both 8 h and 24 h of submergence stress were randomly selected for qRT-PCR using the orchardgrass actin gene as an internal reference gene [53,54]. Primers are listed in Table S8. RNA was reverse-transcribed into cDNA using a PrimeScript ™ RT reagent kit with a gDNA Eraser kit (Takara, Dalian, China), and qRT-PCR was performed with a TB Green Premix Ex TaqTM II kit (Takara, Dalian, China). Three technical replicates were performed for each target gene. The total volume for qRT-PCR was 10 μL with the following procedure: one cycle at 95 °C for 30 s; 40 cycles at 95 °C for 5 s and 60 °C for 34 s; then, 95 °C for 15 s; 60 °C for 1 min; and 95 °C for 15 s. Finally, the relative gene expression was calculated using 2^−ΔΔCt^ [55].

### 4.4. Metabolomics Data Analysis

Root tissues for metabolomics at different times of sampling were the same batch as those used for the transcriptome. A total of six biological replicates of each treatment were used for metabolomics analysis. Approximately 0.1 g of ground root tissue was placed in a tube with 500 μL of 80% aqueous methanol solution. The extraction was shaken and placed in an ice-water bath for 5 min, and then centrifuged at 15,000 r for 20 min at 4 °C. A certain amount of supernatant was taken and diluted with mass-spectrometry-grade water to make a solution containing 53% methanol. The supernatant was collected after centrifuging again for 20 min and used for liquid chromatography coupled with mass spectrometry (LC-MS) [56]. Briefly, an equal volume of samples was mixed as QC samples and 53% aqueous methanol solution was used as blank samples. The chromatographic conditions were as follows: column, HypesilGoldcolumn (C18), column temperature was 40 °C; flow rate was 0.2 mL/min; mobile phase A in positive ionization mode was 0.1% formic acid; mobile phase B was methanol; mobile phase A in negative ionization mode was 5 mM ammonium acetate (pH 9.0); and mobile phase B was methanol. The elution gradient is shown in Table S9. The mass spectrometry conditions were as follows: spray voltage of 3.5 kV; sheath gas flow rate at 35 psi; aux gas flow rate at 10 L/min; capillary temperature at 320 °C; S-lens RF level at 60; aux gas heater temperature at 350 °C; positive and negative polarity; and MS/MS secondary sweep at 350 °C. The MS/MS secondary scans were data-dependent scans. The reagents and instruments used in the experiment are shown in Tables S10 and S11.

The obtained raw data were imported into CD3.1 software, and each metabolite was screened for charge-to-mass ratio and retention-time parameters. The molecular formula of the metabolite was predicted by ion peaks and compared with the database. The relevant interfering ions were removed using blank samples, and the data were processed to obtain quantitative analysis results. Multivariate statistical methods, such as PCA and PLS-DA, were applied to reveal the differences in metabolic patterns among different treatments. Finally, DEMs were defined by VIP > 1.0, fold change (FC) > 1.2 or FC < 0.833, and *p*-value < 0.05. The biological significance associated with the metabolites was explained by functional analyses, such as metabolic pathways.

### 4.5. Integration of Transcriptomic and Metabolomic Analysis

The relationship between DEGs and DEMs were revealed based on Pearson’s correlation coefficient analysis. All the obtained DEGs and DEMs were mapped to the KEGG pathway database to identify biochemical pathways and signal transduction pathways in which DEGs and DEMs were jointly involved. The common highly expressed pathways were identified for further analysis of the expression patterns and network interactions.

## 5. Conclusions

The results elucidated the molecular mechanisms of submergence tolerance in orchardgrass by exploring expressions of genes and metabolites in the roots under short-term stress conditions. The integrated transcriptomic and metabolomic analysis identified key metabolic pathways in response to stress. The up-regulated flavonoid biosynthesis and the up-regulated amino acids biosynthesis observed in the roots could contribute to submergence tolerance. These pathways could be potential targets for further explorations of the role of these pathways in submergence tolerance. Given the complex nature of submergence stress and potential genotype by environment interaction, the results would be helpful in designing future experiments for the dissection of these pathways in more diverse lines in response to different levels of submergence stress.

## Data Availability

All raw sequence reads are available from the NCBI under the Project PRJNA897027. All metabolomic raw results are available from NCBI under the Project PRJCA013073. All other relevant data were included in the paper and Supplementary Materials.

## Supplementary Materials

The following supporting information can be downloaded at: https://www.mdpi.com/article/10.3390/ijms24032089/s1.
