# Supplementary material for: Integrated Transcriptomic and Metabolomics Analysis of the Root Responses of Orchardgrass to Submergence Stress
Source: Int J Mol Sci. 2023 Jan 20;24(3):2089. doi: 10.3390/ijms24032089 (PMC9916531; doi:10.3390/ijms24032089)
Supplement: Supplementary file 1 [file ijms-24-02089-s001.zip › Revised supplemental tables.docx]

| **Table S1. RNA-Sequencing output statistics** | | | | | | | | |
| --- | --- | --- | --- | --- | --- | --- | --- | --- |
| **Treatment samples** | **Replicates** | **Raw reads** | **Clean reads** | **Base (G)** | **Error rate (%)** | **Q20 (%)** | **Q30 (%)** | **Gccontent (%)** |
| 0h-1 | 1 | 47966242 | 47548828 | 7.13 | 0.02 | 98.1 | 94.41 | 52.78 |
| 0h-2 | 2 | 46669862 | 43507876 | 6.53 | 0.02 | 98 | 94.32 | 54.67 |
| 0h-3 | 3 | 47045166 | 46045806 | 6.91 | 0.02 | 97.99 | 94.28 | 55.83 |
| 8h-1 | 1 | 44414498 | 43616674 | 6.54 | 0.03 | 97.9 | 94.02 | 54.79 |
| 8h-2 | 2 | 47685246 | 46691880 | 7 | 0.03 | 97.8 | 93.87 | 54.3 |
| 8h-3 | 3 | 45459856 | 44590486 | 6.69 | 0.03 | 97.94 | 94.11 | 54.83 |
| 24h-1 | 1 | 41700144 | 40769866 | 6.12 | 0.02 | 98 | 94.29 | 54.46 |
| 24h-2 | 2 | 47400554 | 45978932 | 6.9 | 0.02 | 97.99 | 94.24 | 54.56 |
| 24h-3 | 3 | 45061466 | 43289146 | 6.49 | 0.02 | 98 | 94.24 | 52.4 |

| **Table S2. Comparison results between samples and orchardgrass reference genome** | | | | |
| --- | --- | --- | --- | --- |
| **Sample** | **Total mapping (%)** | **Unique mapping (%)** | **Multi mapping (%)** | **Exon (%)** |
| 0h-1 | 31944066(67.18%) | 30952549(65.1%) | 991517(2.09%) | 4045848150(84.99%) |
| 0h-2 | 30029349(69.02%) | 29008828(66.67%) | 1020521(2.35%) | 3897630765(87.09%) |
| 0h-3 | 31577765(68.58%) | 30611916(66.48%) | 965849(2.1%) | 4120371620(87.54%) |
| 8h-1 | 29686484(68.06%) | 27903299(63.97%) | 1783185(4.09%) | 3643990620(82.35%) |
| 8h-2 | 30476491(65.27%) | 28726058(61.52%) | 1750433(3.75%) | 3711527162(81.71%) |
| 8h-3 | 29341079(65.8%) | 28062560(62.93%) | 1278519(2.87%) | 3697926249(84.55%) |
| 24h-1 | 27274129(66.9%) | 25506051(62.56%) | 1768078(4.34%) | 3277185834(80.62%) |
| 24h-2 | 30917979(67.24%) | 28855192(62.76%) | 2062787(4.49%) | 3731103716(80.96%) |
| 24h-3 | 27978934(64.63%) | 26370621(60.92%) | 1608313(3.72%) | 3285938060(78.8%) |

| **Table S3a. Enrichment table of highly expressed differential genes in key pathways (8h vs 0h)** | | | | | | | | | |
| --- | --- | --- | --- | --- | --- | --- | --- | --- | --- |
| **Gene ID** | **GOterm1** | **GOterm2** | **GOterm3** | **GOterm4** | **GOterm5** | **GOterm6** | **GOterm7** | **GOterm8** | **logFC** |
| DG3C06240 | 0 | 0 | 0 | 0 | 1 | 0 | 0 | 0 | 9.0309116 |
| DG2C06064 | 0 | 0 | 0 | 0 | 1 | 0 | 0 | 0 | 7.7870305 |
| DG2C00197 | 0 | 0 | 0 | 0 | 1 | 0 | 0 | 0 | 7.4974335 |
| DG2C06063 | 0 | 0 | 0 | 0 | 1 | 0 | 0 | 0 | 7.4250733 |
| DG2C06062 | 0 | 0 | 0 | 0 | 1 | 0 | 0 | 0 | 7.411362 |
| DG7C03828 | 0 | 0 | 0 | 1 | 0 | 1 | 1 | 0 | 5.0580607 |
| DG7C02886 | 0 | 0 | 0 | 1 | 0 | 1 | 1 | 0 | 3.7054266 |
| DG5C04649 | 0 | 0 | 0 | 0 | 0 | 0 | 0 | 1 | 3.0424515 |
| DG7C02740 | 0 | 0 | 0 | 1 | 0 | 1 | 1 | 0 | 3.0386445 |
| DG1C02206 | 1 | 1 | 1 | 0 | 0 | 0 | 0 | 0 | 2.3785116 |
| DG1C01508 | 1 | 1 | 0 | 0 | 0 | 0 | 0 | 0 | 2.2963339 |
| DG1C07292 | 0 | 0 | 0 | 1 | 0 | 1 | 1 | 0 | 2.2022045 |
| DG1C00750 | 0 | 0 | 0 | 0 | 0 | 0 | 0 | 1 | 2.2014739 |
| DG1C06604 | 0 | 0 | 0 | 0 | 0 | 0 | 0 | 1 | 2.0973277 |
| DG7C02097 | 0 | 0 | 0 | 0 | 1 | 0 | 0 | 0 | 2.0811802 |
| DG2C03109 | 0 | 0 | 0 | 1 | 0 | 1 | 1 | 0 | 2.070355 |
| DG3C05401 | 1 | 1 | 0 | 0 | 0 | 0 | 0 | 0 | 2.0158538 |
| DG2C06768 | 0 | 0 | 0 | 1 | 0 | 1 | 1 | 0 | 1.2408525 |
| DG6C01886 | 0 | 0 | 0 | 0 | 0 | 0 | 0 | 1 | 1.2106109 |
| DG7C01528 | 1 | 1 | 0 | 0 | 0 | 0 | 0 | 0 | -1.06702 |
| DG2C02949 | 0 | 0 | 0 | 1 | 0 | 1 | 1 | 0 | -1.205683 |
| DG1C06753 | 1 | 1 | 1 | 0 | 0 | 0 | 0 | 0 | -1.233205 |
| DG3C02750 | 0 | 0 | 0 | 1 | 0 | 1 | 1 | 0 | -1.325109 |
| novel.9523 | 0 | 0 | 0 | 1 | 0 | 1 | 1 | 0 | -1.337827 |
| DG1C02867 | 1 | 1 | 1 | 0 | 0 | 0 | 0 | 0 | -1.41196 |
| DG3C02607 | 0 | 0 | 0 | 1 | 0 | 1 | 1 | 0 | -1.448734 |
| DG2C03577 | 1 | 1 | 1 | 0 | 0 | 0 | 0 | 0 | -1.483555 |
| DG3C04059 | 1 | 1 | 1 | 0 | 0 | 0 | 0 | 0 | -1.560968 |
| DG6C03659 | 1 | 1 | 1 | 0 | 0 | 0 | 0 | 0 | -1.562287 |
| DG1C02205 | 1 | 1 | 1 | 0 | 0 | 0 | 0 | 0 | -1.619756 |
| DG5C03653 | 1 | 1 | 0 | 0 | 0 | 0 | 0 | 0 | -1.637464 |
| DG2C05408 | 0 | 0 | 0 | 1 | 0 | 0 | 0 | 0 | -1.637763 |
| DG4C03804 | 0 | 0 | 0 | 0 | 0 | 0 | 0 | 1 | -1.649806 |
| DG7C04368 | 0 | 0 | 0 | 1 | 0 | 0 | 0 | 0 | -1.658026 |
| DG2C04057 | 1 | 1 | 0 | 0 | 0 | 0 | 0 | 0 | -1.661642 |
| DG6C00949 | 0 | 0 | 0 | 0 | 1 | 0 | 0 | 0 | -1.680624 |
| DG4C06134 | 1 | 1 | 1 | 0 | 0 | 0 | 0 | 0 | -1.686779 |
| DG6C05218 | 0 | 0 | 0 | 1 | 0 | 0 | 0 | 0 | -1.696387 |
| DG6C01065 | 1 | 1 | 0 | 0 | 0 | 0 | 0 | 0 | -1.69919 |
| DG1C06651 | 0 | 0 | 0 | 1 | 0 | 0 | 0 | 0 | -1.713416 |
| DG2C02819 | 0 | 0 | 0 | 0 | 0 | 0 | 0 | 1 | -1.72613 |
| DG0C00749 | 0 | 0 | 0 | 0 | 0 | 0 | 0 | 1 | -1.74231 |
| DG3C05368 | 0 | 0 | 0 | 0 | 1 | 0 | 0 | 0 | -1.742756 |
| DG6C04662 | 1 | 1 | 1 | 0 | 0 | 0 | 0 | 0 | -1.744518 |
| DG1C06169 | 0 | 0 | 0 | 0 | 1 | 0 | 0 | 0 | -1.753166 |
| DG2C00820 | 0 | 0 | 0 | 0 | 0 | 0 | 0 | 1 | -1.75776 |
| DG2C04515 | 0 | 0 | 0 | 0 | 0 | 0 | 0 | 1 | -1.761137 |
| DG1C06382 | 0 | 0 | 0 | 0 | 1 | 0 | 0 | 0 | -1.764525 |
| DG1C00460 | 0 | 0 | 0 | 1 | 0 | 0 | 0 | 0 | -1.773693 |
| DG1C00274 | 0 | 0 | 0 | 0 | 1 | 0 | 0 | 0 | -1.778178 |
| DG0C00757 | 0 | 0 | 0 | 0 | 1 | 0 | 0 | 0 | -1.786865 |
| DG5C03512 | 0 | 0 | 0 | 0 | 0 | 0 | 0 | 1 | -1.787764 |
| DG1C05650 | 0 | 0 | 0 | 0 | 1 | 0 | 0 | 0 | -1.802893 |
| DG7C02419 | 0 | 0 | 0 | 0 | 1 | 0 | 0 | 0 | -1.81902 |
| DG6C01253 | 0 | 0 | 0 | 1 | 0 | 0 | 0 | 0 | -1.824831 |
| DG3C05171 | 0 | 0 | 0 | 0 | 0 | 0 | 0 | 1 | -1.843814 |
| DG4C03975 | 0 | 0 | 0 | 0 | 0 | 0 | 0 | 1 | -1.869434 |
| DG3C03716 | 0 | 0 | 0 | 0 | 0 | 0 | 0 | 1 | -1.87582 |
| DG3C00995 | 0 | 0 | 0 | 0 | 0 | 0 | 0 | 1 | -1.895406 |
| DG6C03017 | 0 | 0 | 0 | 0 | 0 | 0 | 0 | 1 | -1.911178 |
| DG5C04318 | 1 | 1 | 1 | 0 | 0 | 0 | 0 | 0 | -1.930185 |
| novel.14633 | 1 | 1 | 1 | 0 | 0 | 0 | 0 | 0 | -2.244622 |
| DG1C00674 | 1 | 1 | 1 | 0 | 0 | 0 | 0 | 0 | -2.284616 |
| DG6C05219 | 0 | 0 | 0 | 1 | 0 | 1 | 1 | 0 | -2.306539 |
| DG3C04147 | 1 | 1 | 1 | 0 | 0 | 0 | 0 | 0 | -2.323198 |
| DG1C01350 | 0 | 0 | 0 | 1 | 0 | 1 | 1 | 0 | -2.38899 |
| DG3C04145 | 1 | 1 | 1 | 0 | 0 | 0 | 0 | 0 | -2.739857 |
| DG2C04170 | 0 | 0 | 0 | 1 | 0 | 1 | 1 | 0 | -2.762827 |
| novel.13309 | 0 | 0 | 0 | 1 | 0 | 1 | 1 | 0 | -2.787448 |
| DG6C02834 | 1 | 1 | 0 | 0 | 0 | 0 | 0 | 0 | -2.846372 |
| DG7C03841 | 0 | 0 | 0 | 1 | 1 | 1 | 1 | 0 | -2.907079 |
| DG7C02150 | 1 | 1 | 1 | 0 | 0 | 0 | 0 | 0 | -3.208155 |
| DG3C05944 | 1 | 1 | 1 | 0 | 0 | 0 | 0 | 0 | -3.425622 |
| DG5C04176 | 1 | 1 | 1 | 0 | 0 | 0 | 0 | 0 | -3.431032 |

| GOterm1 | GO:0034637 | cellular carbohydrate biosynthetic process | | | |  |  |
| --- | --- | --- | --- | --- | --- | --- | --- |
| GOterm2 | GO:0016051 | carbohydrate biosynthetic process |  |  |  |  |  |
| GOterm3 | GO:0030243 | cellulose metabolic process | | |  |  |  |
| GOterm4 | GO:0006979 | response to oxidative stress | | |  |  |  |
| GOterm5 | GO:0050662 | coenzyme binding | |  |  |  |  |
| GOterm6 | GO:0016684 | oxidoreductase activity, acting on peroxide as acceptor | | | | | |
| GOterm7 | GO:0003700 | DNA binding transcription factor activity | | | |  |  |
| GOterm8 | GO:0016209 | antioxidant activity | |  |  |  |  |

| **Table S3b. Enrichment table of highly expressed differential genes in key pathways (24h vs 0h)** | | | | | | | | |
| --- | --- | --- | --- | --- | --- | --- | --- | --- |
| **Gene ID** | **GOterm1** | **GOterm2** | **GOterm3** | **GOterm4** | **GOterm5** | **GOterm6** | **GOterm7** | **logFC** |
| DG3C06240 | 0 | 0 | 0 | 1 | 0 | 0 | 0 | 9.7573148 |
| DG2C06064 | 0 | 0 | 0 | 1 | 0 | 0 | 0 | 7.7711881 |
| DG4C01351 | 0 | 0 | 0 | 0 | 1 | 1 | 0 | 7.6870605 |
| DG3C02082 | 0 | 0 | 1 | 0 | 1 | 1 | 0 | 4.0600721 |
| DG1C02206 | 1 | 1 | 0 | 0 | 0 | 0 | 0 | 4.0311097 |
| DG7C02740 | 0 | 0 | 1 | 0 | 1 | 1 | 0 | 3.8899538 |
| DG1C01508 | 1 | 1 | 0 | 0 | 0 | 0 | 0 | 3.0244561 |
| DG1C03324 | 0 | 0 | 1 | 0 | 1 | 1 | 0 | 2.5530446 |
| DG5C05906 | 0 | 0 | 0 | 1 | 0 | 0 | 0 | 2.421445 |
| DG2C06697 | 1 | 1 | 0 | 0 | 0 | 0 | 0 | 2.359102 |
| DG5C06690 | 0 | 0 | 0 | 0 | 1 | 1 | 0 | 1.3271396 |
| DG1C03572 | 0 | 0 | 1 | 0 | 1 | 1 | 0 | 1.2921696 |
| DG4C01943 | 1 | 1 | 0 | 0 | 0 | 0 | 0 | 1.2213625 |
| DG1C07257 | 0 | 0 | 1 | 0 | 1 | 1 | 0 | 1.1741027 |
| DG6C05009 | 0 | 0 | 1 | 0 | 1 | 1 | 0 | -1.483772 |
| DG5C04434 | 0 | 0 | 0 | 0 | 1 | 1 | 0 | -1.940618 |
| DG7C01528 | 1 | 1 | 0 | 0 | 0 | 0 | 0 | -1.943476 |
| DG3C02850 | 0 | 0 | 0 | 0 | 0 | 0 | 1 | -1.948134 |
| DG3C02607 | 0 | 0 | 1 | 0 | 1 | 1 | 0 | -1.979787 |
| DG3C01317 | 0 | 0 | 0 | 1 | 0 | 0 | 0 | -1.994047 |
| DG1C03705 | 0 | 0 | 1 | 0 | 1 | 1 | 0 | -2.002423 |
| DG6C03659 | 1 | 1 | 0 | 0 | 0 | 0 | 0 | -2.119891 |
| DG3C00258 | 0 | 0 | 0 | 0 | 1 | 1 | 0 | -2.129004 |
| DG4C03045 | 0 | 0 | 1 | 0 | 1 | 1 | 0 | -2.155697 |
| DG6C04662 | 1 | 1 | 0 | 0 | 0 | 0 | 0 | -2.205809 |
| DG1C06803 | 0 | 0 | 1 | 0 | 1 | 1 | 0 | -2.224759 |
| DG7C04798 | 0 | 0 | 0 | 1 | 0 | 0 | 0 | -2.241529 |
| DG6C01253 | 0 | 0 | 1 | 0 | 1 | 1 | 0 | -2.24473 |
| DG3C01162 | 0 | 0 | 0 | 1 | 0 | 0 | 0 | -2.297242 |
| DG2C05408 | 0 | 0 | 1 | 0 | 1 | 1 | 0 | -2.326086 |
| DG6C02834 | 1 | 1 | 0 | 0 | 0 | 0 | 0 | -2.395153 |
| novel.6339 | 0 | 0 | 0 | 1 | 0 | 0 | 0 | -2.425972 |
| DG1C06382 | 0 | 0 | 0 | 1 | 0 | 0 | 0 | -2.433858 |
| DG2C03577 | 1 | 1 | 0 | 0 | 0 | 0 | 0 | -2.4362 |
| DG2C06724 | 0 | 0 | 0 | 1 | 0 | 0 | 0 | -2.50317 |
| DG1C02205 | 1 | 1 | 0 | 0 | 0 | 0 | 0 | -2.504914 |
| DG1C00674 | 1 | 1 | 0 | 0 | 0 | 0 | 0 | -2.52098 |
| DG4C04265 | 0 | 0 | 0 | 1 | 0 | 0 | 0 | -2.532254 |
| DG3C02602 | 0 | 0 | 1 | 0 | 1 | 1 | 0 | -2.552755 |
| DG6C00949 | 0 | 0 | 0 | 1 | 0 | 0 | 0 | -2.592332 |
| novel.14632 | 1 | 1 | 0 | 0 | 0 | 0 | 0 | -2.616115 |
| novel.13309 | 0 | 0 | 1 | 0 | 1 | 1 | 0 | -2.626276 |
| DG5C02092 | 0 | 0 | 1 | 0 | 1 | 1 | 0 | -2.636034 |
| DG5C04971 | 0 | 0 | 0 | 1 | 0 | 0 | 0 | -2.657871 |
| DG6C01065 | 1 | 1 | 0 | 0 | 0 | 0 | 0 | -2.710881 |
| DG5C02094 | 0 | 0 | 1 | 0 | 1 | 1 | 0 | -2.722188 |
| DG5C04293 | 1 | 0 | 0 | 0 | 0 | 0 | 0 | -2.849755 |
| DG1C01404 | 0 | 0 | 0 | 1 | 0 | 0 | 0 | -2.85498 |
| DG6C05229 | 0 | 0 | 1 | 0 | 1 | 1 | 0 | -2.859664 |
| DG2C05014 | 0 | 0 | 0 | 1 | 0 | 0 | 0 | -2.901528 |
| DG5C04526 | 0 | 0 | 0 | 0 | 1 | 1 | 0 | -2.902447 |
| DG5C04318 | 1 | 1 | 0 | 0 | 0 | 0 | 0 | -2.917167 |
| DG2C03442 | 0 | 0 | 0 | 0 | 1 | 1 | 0 | -3.002017 |
| DG7C02150 | 1 | 1 | 0 | 0 | 0 | 0 | 0 | -3.066501 |
| DG1C01351 | 0 | 0 | 1 | 0 | 1 | 1 | 0 | -3.139033 |
| DG4C01238 | 1 | 1 | 0 | 0 | 0 | 0 | 0 | -3.174599 |
| DG3C01234 | 0 | 0 | 0 | 1 | 0 | 0 | 0 | -3.203059 |
| DG1C00460 | 0 | 0 | 1 | 0 | 1 | 1 | 0 | -3.228815 |
| DG0C00094 | 1 | 1 | 0 | 0 | 0 | 0 | 0 | -3.386668 |
| DG7C04368 | 0 | 0 | 1 | 0 | 1 | 1 | 0 | -3.398413 |
| DG7C03841 | 0 | 0 | 1 | 0 | 1 | 1 | 0 | -3.409201 |
| DG3C04059 | 1 | 1 | 0 | 0 | 0 | 0 | 0 | -3.445115 |
| DG3C02608 | 0 | 0 | 1 | 0 | 1 | 1 | 0 | -3.45842 |
| DG3C05944 | 1 | 1 | 0 | 0 | 0 | 0 | 0 | -3.461487 |
| DG2C04163 | 0 | 0 | 1 | 0 | 1 | 1 | 0 | -4.28533 |
| DG3C04147 | 1 | 1 | 0 | 0 | 0 | 0 | 0 | -4.731923 |
| DG2C04166 | 0 | 0 | 1 | 0 | 1 | 1 | 0 | -5.247604 |
| DG2C02047 | 0 | 0 | 0 | 1 | 0 | 0 | 0 | -5.265636 |
| DG7C00925 | 0 | 0 | 1 | 0 | 1 | 1 | 0 | -5.452199 |
| DG7C01254 | 0 | 0 | 0 | 1 | 0 | 0 | 0 | -5.889018 |
| DG6C05007 | 0 | 0 | 1 | 0 | 1 | 1 | 0 | -5.924314 |
| DG3C07173 | 0 | 0 | 0 | 1 | 0 | 0 | 0 | -6.809163 |
| DG2C07101 | 0 | 0 | 1 | 0 | 1 | 1 | 0 | -6.979169 |
| DG4C02594 | 0 | 0 | 0 | 1 | 0 | 0 | 0 | -7.088888 |
| DG3C05683 | 0 | 0 | 1 | 0 | 1 | 1 | 0 | -7.159251 |
| DG3C07331 | 0 | 0 | 0 | 1 | 0 | 0 | 0 | -7.892317 |

| GOterm1 | GO:0016051 | carbohydrate biosynthetic process | | |
| --- | --- | --- | --- | --- |
| GOterm2 | GO:0034637 | carbohydrate biosynthetic process | | |
| GOterm3 | GO:0006979 | response to oxidative stress | |  |
| GOterm4 | GO:0050662 | coenzyme binding |  |  |
| GOterm5 | GO:0004601 | peroxidase activity |  |  |
| GOterm6 | GO:0016209 | antioxidant activity |  |  |
| GOterm7 | GO:0005798 | Golgi-associated vesicle | |  |

| **Table S4a. Statistical table of the number of DEGs in each pathway (8h vs 0h)** | | | | | | |
| --- | --- | --- | --- | --- | --- | --- |
| **KEGGID** | **Description** | **P value** | **padj** | **Count** | **Down** | **Up** |
| ath00940 | Phenylpropanoid biosynthesis | 1.48E-13 | 1.79E-11 | 124 | 109 | 15 |
| ath00010 | Glycolysis / Gluconeogenesis | 3.83E-06 | 0.000231647 | 59 | 17 | 42 |
| ath00941 | Flavonoid biosynthesis | 9.20E-06 | 0.000371105 | 30 | 28 | 2 |
| ath00500 | Starch and sucrose metabolism | 4.66E-05 | 0.001189584 | 65 | 42 | 23 |
| ath00400 | Phenylalanine, tyrosine and tryptophan biosynthesis | 4.92E-05 | 0.001189584 | 27 | 24 | 3 |
| ath01230 | Biosynthesis of amino acids | 0.000137529 | 0.002464714 | 84 | 45 | 39 |
| ath04016 | MAPK signaling pathway - plant | 0.000142587 | 0.002464714 | 61 | 38 | 23 |
| ath00270 | Cysteine and methionine metabolism | 0.000217593 | 0.003291095 | 45 | 26 | 19 |
| ath00360 | Phenylalanine metabolism | 0.000339301 | 0.004561709 | 24 | 20 | 4 |
| ath04626 | Plant-pathogen interaction | 0.000441472 | 0.005341816 | 76 | 53 | 23 |
| ath04075 | Plant hormone signal transduction | 0.000517387 | 0.005691253 | 85 | 55 | 30 |
| ath00260 | Glycine, serine and threonine metabolism | 0.001056648 | 0.010654532 | 27 | 17 | 10 |
| ath00520 | Amino sugar and nucleotide sugar metabolism | 0.001386971 | 0.012102995 | 53 | 38 | 15 |
| ath00945 | Stilbenoid, diarylheptanoid and gingerol biosynthesis | 0.001477785 | 0.012102995 | 14 | 12 | 2 |
| ath00592 | alpha-Linolenic acid metabolism | 0.001500371 | 0.012102995 | 29 | 16 | 13 |
| ath00350 | Tyrosine metabolism | 0.001840834 | 0.01392131 | 20 | 12 | 8 |
| ath00460 | Cyanoamino acid metabolism | 0.002415433 | 0.017192202 | 26 | 22 | 4 |
| ath00650 | Butanoate metabolism | 0.003764713 | 0.025307234 | 12 | 9 | 3 |
| ath00944 | Flavone and flavonol biosynthesis | 0.00457375 | 0.029127563 | 9 | 7 | 2 |
| ath00910 | Nitrogen metabolism | 0.007421305 | 0.043135805 | 16 | 10 | 6 |
| ath00591 | Linoleic acid metabolism | 0.007486379 | 0.043135805 | 8 | 6 | 2 |
|  |  |  |  |  |  |  |
| **Table S4b. Statistical table of the number of differential genes in each pathway (24h vs 0h)** | | | | | | |
| **KEGGID** | **Description** | **P value** | **padj** | **Count** | **Down** | **Up** |
| ath00010 | Glycolysis / Gluconeogenesis | 1.55E-09 | 1.89E-07 | 85 | 38 | 47 |
| ath00940 | Phenylpropanoid biosynthesis | 1.26E-06 | 5.58548E-05 | 136 | 117 | 19 |
| ath00400 | Phenylalanine, tyrosine and tryptophan biosynthesis | 1.37E-06 | 5.58548E-05 | 37 | 31 | 6 |
| ath01200 | Carbon metabolism | 5.75E-06 | 0.000175299 | 122 | 67 | 55 |
| ath00030 | Pentose phosphate pathway | 8.23E-06 | 0.000200738 | 33 | 15 | 18 |
| ath04075 | Plant hormone signal transduction | 1.46635E-05 | 0.000298158 | 121 | 74 | 47 |
| ath00620 | Pyruvate metabolism | 2.39333E-05 | 0.000417122 | 46 | 26 | 20 |
| ath01230 | Biosynthesis of amino acids | 5.76232E-05 | 0.000878755 | 114 | 68 | 46 |
| ath00051 | Fructose and mannose metabolism | 0.000418516 | 0.005216903 | 34 | 14 | 20 |
| ath00630 | Glyoxylate and dicarboxylate metabolism | 0.000427615 | 0.005216903 | 37 | 23 | 14 |
| ath00910 | Nitrogen metabolism | 0.000511768 | 0.005675974 | 22 | 17 | 5 |
| ath00500 | Starch and sucrose metabolism | 0.001185795 | 0.012055582 | 80 | 54 | 26 |
| ath00650 | Butanoate metabolism | 0.003503492 | 0.032878926 | 15 | 12 | 3 |
| ath04626 | Plant-pathogen interaction | 0.004722014 | 0.041148983 | 96 | 67 | 29 |
| ath00270 | Cysteine and methionine metabolism | 0.005492748 | 0.044674352 | 53 | 32 | 21 |
| ath00053 | Ascorbate and aldarate metabolism | 0.00614127 | 0.045104039 | 27 | 20 | 7 |
| ath00460 | Cyanoamino acid metabolism | 0.006284989 | 0.045104039 | 32 | 27 | 5 |
|  |  |  |  |  |  |  |

| **Table S5a. Statistical table of the number of DEGs in each pathway (8h vs 0h)** | | | | | |
| --- | --- | --- | --- | --- | --- |
| **Map ID** | **Map Title** | **P value** | **x** | **y** | **Meta IDs** |
| map00053 | Ascorbate and aldarate metabolism | 0.011324485 | 3 | 3 | alpha-Ketoglutaric acid; D-Galacturonic acid; |
|  |  |  |  |  | D-Saccharic acid |
| map00770 | Pantothenate and CoA biosynthesis | 0.038053601 | 3 | 4 | Pantothenic acid; Panthenol; Uracil |
| map00040 | Pentose and glucuronate interconversions | 0.05171515 | 2 | 2 | alpha-Ketoglutaric acid; D-Galacturonic acid |
| map00380 | Tryptophan metabolism | 0.05171515 | 2 | 2 | Indole; Tryptophan |
| map01210 | 2-Oxocarboxylic acid metabolism | 0.080028213 | 3 | 5 | L-Ornithine; Citraconic acid; Tryptophan |
| map00410 | beta-Alanine metabolism | 0.132496478 | 2 | 3 | Pantothenic acid; Uracil |
| map00220 | Arginine biosynthesis | 0.134845804 | 3 | 6 | alpha-Ketoglutaric acid; L-Ornithine; Fumaric acid |
| map00400 | Phenylalanine, tyrosine and tryptophan biosynthesis | 0.134845804 | 3 | 6 | Shikimic acid; Indole; Tryptophan |
| map00941 | Flavonoid biosynthesis | 0.176265254 | 5 | 13 | Apigenin; Naringenin; Hesperetin; |
|  |  |  |  |  | Naringenin chalcone; Liquiritigenin |
| map00020 | Citrate cycle (TCA cycle) | 0.226939355 | 2 | 4 | alpha-Ketoglutaric acid; Fumaric acid |
| map00350 | Tyrosine metabolism | 0.226939355 | 2 | 4 | Fumaric acid; Salidroside |
| map00520 | Amino sugar and nucleotide sugar metabolism | 0.226939355 | 2 | 4 | D-Galacturonic acid; D-Mannose 6-phosphate |
| map00650 | Butanoate metabolism | 0.226939355 | 2 | 4 | alpha-Ketoglutaric acid; Fumaric acid |
| map00660 | C5-Branched dibasic acid metabolism | 0.226939355 | 2 | 4 | alpha-Ketoglutaric acid; Citraconic acid |
| map00051 | Fructose and mannose metabolism | 0.230215827 | 1 | 1 | D-Mannose 6-phosphate |
| map00290 | Valine, leucine and isoleucine biosynthesis | 0.230215827 | 1 | 1 | Citraconic acid |
| map00790 | Folate biosynthesis | 0.230215827 | 1 | 1 | Sepiapterin |
| map01040 | Biosynthesis of unsaturated fatty acids | 0.385168416 | 3 | 8 | Nervonic acid; Arachidonic acid; Adrenic acid |
| map01100 | Metabolic pathways | 0.395869979 | 21 | 80 | Apigenin; Ferulaldehyde; Arachidonic acid; UDP; |
|  | | | | | D-Saccharic acid; Fumaric acid; Pantothenic acid; |
|  |  |  |  |  | Adenosine;Citraconic acid;Indole;Naringenin chalcone; |
|  |  |  |  |  | Tryptophan;Trehalose 6-phosphate;Phytosphingosine; |
|  |  |  |  |  | D-Galacturonic acid; L-Ornithine;Naringenin; Shikimic acid; |
|  |  |  |  |  | Jasmonic acid; D-Mannose 6-phosphate; Uracil; |
| map00052 | Galactose metabolism | 0.408716505 | 1 | 2 | Stachyose |
|  |  |  |  |  |  |
| **Table S5b. Statistical table of the number of DEGs in each pathway (24h vs 0h)** | | | | | |
| **Map ID** | **Map Title** | **P value** | **x** | **y** | **Meta IDs** |
| map00300 | Lysine biosynthesis | 0.077259931 | 2 | 2 | L-Saccharopine; alpha-Ketoglutaric acid |
| map00380 | Tryptophan metabolism | 0.077259931 | 2 | 2 | Indole; Tryptophan |
| map00640 | Propanoate metabolism | 0.077259931 | 2 | 2 | Succinic acid; Methylmalonic acid |
| map01210 | 2-Oxocarboxylic acid metabolism | 0.134400434 | 3 | 5 | Citraconic acid; Tryptophan; N-Acetyl-L-glutamate |
| map00053 | Ascorbate and aldarate metabolism | 0.190048152 | 2 | 3 | alpha-Ketoglutaric acid; D-Saccharic acid |
| map00310 | Lysine degradation | 0.190048152 | 2 | 3 | L-Saccharopine; 5-Aminovaleric acid |
| map00592 | alpha-Linolenic acid metabolism | 0.190048152 | 2 | 3 | 13(S)-HOTrE; Jasmonic acid |
| map00941 | Flavonoid biosynthesis | 0.190814565 | 6 | 13 | Apigenin; Neohesperidin; Naringenin; |
|  |  |  |  |  | Naringin; Naringenin chalcone; Liquiritigenin |
| map00061 | Fatty acid biosynthesis | 0.28057554 | 1 | 1 | Oleic Acid |
| map00073 | Cutin, suberine and wax biosynthesis | 0.28057554 | 1 | 1 | Oleic Acid |
| map00280 | Valine, leucine and isoleucine degradation | 0.28057554 | 1 | 1 | Methylmalonic acid |
| map00290 | Valine, leucine and isoleucine biosynthesis | 0.28057554 | 1 | 1 | Citraconic acid |
| map00670 | One carbon pool by folate | 0.28057554 | 1 | 1 | 10-Formyl-THF |
| map00790 | Folate biosynthesis | 0.28057554 | 1 | 1 | Sepiapterin |
| map00920 | Sulfur metabolism | 0.28057554 | 1 | 1 | Succinic acid |
| map00020 | Citrate cycle (TCA cycle) | 0.313202937 | 2 | 4 | alpha-Ketoglutaric acid; Succinic acid |
| map00270 | Cysteine and methionine metabolism | 0.313202937 | 2 | 4 | Glutathione; 5'-Methylthioadenosine |
| map00630 | Glyoxylate and dicarboxylate metabolism | 0.313202937 | 2 | 4 | alpha-Ketoglutaric acid; Succinic acid |
| map00650 | Butanoate metabolism | 0.313202937 | 2 | 4 | alpha-Ketoglutaric acid; Succinic acid |
| map00660 | C5-Branched dibasic acid metabolism | 0.313202937 | 2 | 4 | alpha-Ketoglutaric acid; Citraconic acid |

**Table S6a. Top 20 differential metabolites functional enrichment table (8h vs 0h)**

| **Name** | **Molecular Weight** | **RT [min]** | **KEGG pathway** | **Sub Class (HMDB)** | **Sub Class**  **(Lipid maps)** |
| --- | --- | --- | --- | --- | --- |
| Tryptophan | 204.08979 | 6.763 | Biosynthesis of secondary metabolites;2-Oxocarboxylic acid metabolism; | Indolyl carboxylic acids and derivatives | -- |
|  |  |  | Glycine, serine and threonine metabolism; | | |
|  |  |  | Phenylalanine, tyrosine and tryptophan biosynthesis; | | |
|  |  |  | Indole alkaloid biosynthesis;Glucosinolate biosynthesis; | | |
|  |  |  | Aminoacyl-tRNA biosynthesis;Metabolic pathways; | | |
|  |  |  | Biosynthesis of amino acids;Tryptophan metabolism; | | |
| R-Aminobutyrate | 103.06381 | 1.163 | -- | -- | -- |
| Pantothenic acid | 219.11061 | 6.419 | beta-Alanine metabolism;  Pantothenate and CoA biosynthesis; | Polyols | -- |
|  |  |  | Metabolic pathways;  Biosynthesis of secondary metabolites | | |
|  |  |  |  |  |  |
| Naringenin chalcone | 272.06829 | 10.985 | Flavonoid biosynthesis; Metabolic pathways; | Chalcones and dihydrochalcones | Chalcones and dihydrochalcones |
|  |  |  | Biosynthesis of secondary metabolites | | |
| C-hexosyl-chrysoeriol O-feruloylhexoside | 800.21377 | 10.32 | -- | -- | -- |
| Gamma-Aminobutyric acid | 103.06378 | 1.678 | -- | Amino acids, peptides, and analogues | -- |
| 3-Hydroxy-9,10-Dimethoxypterocarpan | 300.09948 | 9.189 | -- | Furanoisoflavonoids | Pterocarpans |
| L-Cysteine-glutathione gisulfide | 426.08672 | 1.299 | -- | -- | -- |
| LysoPC 16:2 | 491.30065 | 14.769 | -- | -- | -- |
| N-[(-)-Jasmonoyl]-(L)-Isoleucine | 323.20937 | 12.43 | -- | -- | -- |
| N-Coumaroyltyramine | 283.12064 | 10.005 | -- | -- | -- |
| D-(-)-Ribose | 150.05292 | 1.281 | -- | -- | -- |
| Jasmonic acid | 210.12567 | 9.879 | alpha-Linolenic acid metabolism;  Metabolic pathways; | Lineolic acids and derivatives | Jasmonic acids |
|  |  |  | Biosynthesis of secondary metabolites;  Plant hormone signal transduction | | |
| DGDG (18:2/18:3) | 998.62192 | 15.187 | -- | -- | -- |
| 2-(Dimethylamino)Guanosine | 311.12172 | 1.335 | -- | -- | -- |
| N-Sinapoylagmatine | 336.1785 | 9.375 | -- | -- | -- |
| N-lactoyl-phenylalanine | 237.10006 | 8.243 | -- | -- | -- |
| Dihydrojasmone | 166.13585 | 9.892 | -- | Ketones | -- |
| Alisol B | 472.35521 | 15.98 | -- | -- | -- |
|  |  |  | Biosynthesis of secondary metabolites | | |
| Naringenin | 272.06822 | 10.87 | Flavonoid biosynthesis;Metabolic pathways; | Flavans | Flavanones |
|  |  |  |  |  |  |
| **Table S6b. Top20 differential metabolites functional enrichment table (24h vs 0h)** | | | | | |
| **Name** | **Molecular Weight** | **RT [min]** | **KEGG pathway** | **Sub Class (HMDB)** | **Sub Class**  **(Lipidmaps)** |
| R-Aminobutyrate | 103.06381 | 1.163 | -- | -- | -- |
| Naringenin chalcone | 272.06829 | 10.985 | Flavonoid biosynthesis;Metabolic pathways; | Chalcones and dihydrochalcones | Chalcones and dihydrochalcones |
|  |  |  | Biosynthesis of secondary metabolites | | |
| N-epsilon-Acetyllysine | 188.11317 | 1.351 | -- | Amino acids, peptides, and analogues | -- |
| Gamma-Aminobutyric acid | 103.06378 | 1.678 | -- | Amino acids, peptides, and analogues | -- |
| Kinsenoside | 264.08409 | 1.47 | -- | -- | -- |
| Panthenol | 205.13133 | 6.493 | Pantothenate and CoA biosynthesis | -- | -- |
| DHZROG | 515.22099 | 9.51 | -- | -- | -- |
| 5-O-p-Coumaroyl shikimic acid | 320.08934 | 9.215 | -- | -- | -- |
| N-[(-)-Jasmonoyl]-(L)-Isoleucine | 323.20937 | 12.43 | -- | -- | -- |
| Apigenin C-pentoside | 402.0946 | 10.122 | -- | -- | -- |
| Jasmonic acid | 210.12567 | 9.879 | alpha-Linolenic acid metabolism; Metabolic pathways; | Lineolic acids and derivatives | Jasmonic acids |
|  |  |  | Biosynthesis of secondary metabolites;  Plant hormone signal transduction | | |
| Apigenin | 270.05259 | 11.451 | Flavonoid biosynthesis;  Flavone and flavonol biosynthesis; | Flavones | Flavones and Flavonols |
|  |  |  | Metabolic pathways;  Biosynthesis of secondary metabolites | | |
| Naringenin | 272.06822 | 10.87 | Flavonoid biosynthesis;Metabolic pathways; | Flavans | Flavanones |
|  |  |  | Biosynthesis of secondary metabolites | | |
| Phenylacetaldehyde | 120.05764 | 6.358 | Phenylalanine metabolism;Metabolic pathways | _ | -- |
| Caffeic aldehyde | 164.04721 | 6.363 | Phenylpropanoid biosynthesis;Biosynthesis of secondary metabolites | _ | -- |
| Heterophyllin B | 778.43501 | 15.927 | -- | -- | -- |
| Purpureaside C | 786.26193 | 6.342 | -- | -- | -- |
| Scutellarin | 462.07995 | 6.354 | -- | -- | -- |
| N-lactoyl-phenylalanine | 237.10006 | 8.243 | -- | -- | -- |
| Sepiapterin | 237.08575 | 11.297 | Folate biosynthesis | Pterins and derivatives | -- |
| Dihydrojasmone | 166.13585 | 9.892 | -- | Ketones | -- |

**Table S7a. Statistical table of the number of DEGs in biosynthesis of flavonoid and amino acids**

| **KEGG Pathway** | **Gene ID** | **DB 8h vs 0h log2FC** | **DB 24h vs 0h log2FC** | **Description** |
| --- | --- | --- | --- | --- |
| Flavonoid biosynthesis | DG3C07254 | -4.45434 | -4.50734 | PF01596:O-methyltransferase |
|  | DG7C03073 | -4.76387 | -6.17832 | PF00067: Cytochrome P450 |
|  | DG2C03381 | -7.4536 | -9.14304 | PF00195: Chalcone and stilbene synthases, N-terminal domain\|  PF02797: Chalcone and stilbene synthases, C-terminal domain |
|  | DG5C04650 | -4.24466 | -4.15394 | PF00067: Cytochrome P450 |
|  | DG2C04174 | -2.96809 | -4.54541 | PF00067: Cytochrome P450 |
|  | DG1C06378 | -3.89816 | -5.98757 | PF01370: NAD dependent epimerase/dehydratase family |
|  | DG3C05816 | -3.12004 | -3.78275 | PF00067: Cytochrome P450 |
|  | DG3C02730 | -5.23281 | -6.23827 | PF02431: Chalcone-flavanone isomerase |
|  | DG2C02162 | 2.988825 | 3.587701 | PF00067: Cytochrome P450 |
|  | novel.9509 | -2.41403 | -2.74367 | PF00067: Cytochrome P450 |
|  | DG1C06379 | -5.87195 | -5.11549 | PF01370: NAD dependent epimerase/dehydratase family |
|  | DG7C03078 | -3.56265 | -4.90845 | PF00067: Cytochrome P450 |
|  | DG6C05738 | -5.36053 | -8.80184 | PF02458: Transferase family |
|  | DG4C05841 | -2.00763 | -2.83106 | PF02431: Chalcone-flavanone isomerase |
|  | DG3C07154 | -2.08106 | -3.61693 | PF02458: Transferase family |
|  | DG2C06951 | -2.37201 | -4.89942 | PF02458: Transferase family |
|  | DG1C06382 | -1.76452 | -2.43386 | PF01370: NAD dependent epimerase/dehydratase family |
|  | DG3C00468 | -1.38902 | -2.02516 | PF02458: Transferase family |
|  | DG1C05519 | -1.69018 | -2.15937 | PF02458: Transferase family |
|  | DG3C04489 | -1.90307 | -2.23282 | PF00067: Cytochrome P450 |
|  | DG3C04490 | -1.73592 | -1.90997 | PF00067: Cytochrome P450 |
|  | DG3C07153 | -1.66772 | -2.19776 | PF02458: Transferase family |
|  | DG3C04057 | 0.846792 | 1.069567 | PF02458: Transferase family |
|  | DG3C07159 | -5.8734 | -3.43471 | PF02458: Transferase family |
|  | DG3C07172 | -4.21648 | -3.68056 | PF02458: Transferase family |
|  | DG4C00902 | -4.3074 | -3.46941 | PF02458: Transferase family |
|  | DG1C06381 | -0.97331 | -1.28991 | PF01370: NAD dependent epimerase/dehydratase family |
|  | DG3C06963 | -2.51102 | -1.68053 | PF00195: Chalcone and stilbene synthases, N-terminal domain\|  PF02797: Chalcone and stilbene synthases, C-terminal domain |
|  | DG3C05616 | -0.35495 | -1.25257 | PF01596:O-methyltransferase |
| Biosynthesis of amino acids | DG6C00996 | 7.82348627 | 8.082985 | PF00365: Phosphofructokinase |
|  | DG5C05064 | 5.61234388 | 6.533134 | PF00121: Triosephosphate isomerase |
|  | DG4C06263 | 4.38471462 | 5.717562 | PF01842: ACT domain\|  PF00696: Amino acid kinase family |
|  | DG5C05662 | 4.19620579 | 4.734044 | PF00274: Fructose-bisphosphate aldolase class-I |
|  | DG6C03895 | 4.11916397 | 3.986894 | PF00155: Aminotransferase class I and II |
|  | DG2C01004 | 3.91100682 | 4.248531 | PF00300: Histidine phosphatase superfamily (branch 1) |
|  | DG2C02324 | 3.89379689 | 3.718584 | PF00162: Phosphoglycerate kinase |
|  | DG5C03746 | 3.25928427 | 3.422858 | PF01645: Conserved region in glutamate synthase\|  PF14691: Dihydroprymidine dehydrogenase domain II, 4Fe-4S cluster\|  PF01493: GXGXG motif\|  PF07992: Pyridine nucleotide-disulphide oxidoreductase\|  PF04898: Glutamate synthase central domain\|  PF00310: Glutamine amidotransferases class-II |
|  | DG4C03771 | 3.19239827 | 3.92296 | PF02774: Semialdehyde dehydrogenase, dimerisation domain\|  PF01118: Semialdehyde dehydrogenase, NAD binding domain |
|  | DG5C04203 | 3.11590658 | 3.16774 | PF00046: Homeobox domain\|  PF00155: Aminotransferase class I and II\|  PF01852: START domain |
|  | DG3C02444 | 3.08278999 | 3.498575 | PF02887: Pyruvate kinase, alpha/beta domain\|  PF00224: Pyruvate kinase, barrel domain |
|  | DG1C07053 | 2.79873263 | 2.967335 | PF00224: Pyruvate kinase, barrel domain\|  PF02887: Pyruvate kinase, alpha/beta domain |
|  | DG3C01167 | 2.74403543 | 3.521951 | PF00044: Glyceraldehyde 3-phosphate dehydrogenase, NAD binding domain\|  PF02800: Glyceraldehyde 3-phosphate dehydrogenase, C-terminal domain |
|  | DG6C04544 | 2.73158141 | 3.711105 | PF00365: Phosphofructokinase |
|  | DG0C00073 | 2.4093939 | 2.073726 | PF00696: Amino acid kinase family\|  PF01842: ACT domain\|  PF03447: Homoserine dehydrogenase, NAD binding domain\|  PF00742: Homoserine dehydrogenase |
|  | DG3C06168 | 2.36077584 | 2.711043 | PF03952: Enolase, N-terminal domain\|  PF00113: Enolase, C-terminal TIM barrel domain |
|  | DG7C00863 | 2.24880912 | 3.209465 | PF00920: Dehydratase family |
|  | DG2C05318 | 2.13664655 | 2.607227 | PF00120: Glutamine synthetase, catalytic domain\|  PF03951: Glutamine synthetase, beta-Grasp domain |
|  | DG2C04597 | 2.09250795 | 2.382318 | PF02887: Pyruvate kinase, alpha/beta domain\|  PF00224: Pyruvate kinase, barrel domain |
|  | DG2C05592 | -2.0219583 | -2.70864 | PF01264: Chorismate synthase |
|  | DG1C04162 | -2.1015724 | -2.70849 | PF00800: Prephenate dehydratase |
|  | DG3C06814 | -2.2451061 | -3.21544 | PF00300: Histidine phosphatase superfamily (branch 1) |
|  | DG1C00461 | -2.2647572 | -3.15517 | PF00800: Prephenate dehydratase |
|  | DG5C02432 | -2.2710751 | -2.64665 | PF02773:S-adenosylmethionine synthetase, C-terminal domain\|  PF02772:S-adenosylmethionine synthetase, central domain\|  PF00438:S-adenosylmethionine synthetase, N-terminal domain |
|  | DG5C02431 | -2.2844194 | -2.68526 | PF02772:S-adenosylmethionine synthetase, central domain\|  PF00438:S-adenosylmethionine synthetase, N-terminal domain\|  PF02773:S-adenosylmethionine synthetase, C-terminal domain |
|  | DG1C01528 | -2.535279 | -3.40649 | PF01474: Class-II DAHP synthetase family |
|  | DG1C05879 | -2.6072337 | -2.60694 | PF00117: Glutamine amidotransferase class-I |
|  | DG3C06803 | -3.1676828 | -3.92943 | PF01202: Shikimate kinase |
|  | DG4C04775 | -3.3138212 | -4.7393 | PF08267: Cobalamin-independent synthase, N-terminal domain\|  PF01717: Cobalamin-independent synthase, Catalytic domain |
|  | DG2C05691 | -3.3270799 | -3.4068 | PF00425: chorismate binding enzyme\|  PF04715: Anthranilate synthase component I, N terminal region |
|  | DG7C01254 | -3.3675791 | -5.88902 | PF02826:D-isomer specific 2-hydroxyacid dehydrogenase, NAD binding domain\|  PF01842: ACT domain\|PF00389:D-isomer specific 2-hydroxyacid dehydrogenase, catalytic domain |
|  | DG3C03188 | -3.457657 | -4.03252 | PF00218: Indole-3-glycerol phosphate synthase |
|  | DG2C03542 | -3.5085025 | -4.82904 | PF00464: Serine hydroxymethyltransferase |
|  | DG1C05059 | -3.8792128 | -4.24253 | PF01053: Cys/Met metabolism PLP-dependent enzyme |
|  | DG2C05014 | -3.9156365 | -2.90153 | PF00155: Aminotransferase class I and II |
|  | DG2C04022 | -4.2693158 | -6.03533 | PF01474: Class-II DAHP synthetase family |
|  | DG1C05058 | -4.556591 | -4.45362 | PF01053: Cys/Met metabolism PLP-dependent enzyme |
|  | DG2C04021 | -5.0243476 | -6.44071 | PF01474: Class-II DAHP synthetase family |
|  | DG4C05484 | -5.3446802 | -6.66913 | PF00290: Tryptophan synthase alpha chain |

**Table S7b. Statistical table of the number of DEMs in biosynthesis of flavonoid and amino acids**

| **KEGG Pathway** | **Name** | **RT [min]** | **Molecular Weight** | **KEGG Map** |
| --- | --- | --- | --- | --- |
| Flavonoid biosynthesis | Naringenin | 10.87 | 272.0682 | Flavonoid biosynthesis; Metabolic pathways; Biosynthesis of secondary metabolites |
|  | Apigenin | 11.451 | 270.0526 | Flavonoid biosynthesis; Flavone and flavonol biosynthesis; Metabolic pathways; Biosynthesis of secondary metabolites |
|  | Naringin | 9.603 | 580.18 | Flavonoid biosynthesis |
|  | Neohesperidin | 9.659 | 610.1903 | Flavonoid biosynthesis |
|  | Liquiritigenin | 11.529 | 256.0733 | Flavonoid biosynthesis; Biosynthesis of secondary metabolites |
| Biosynthesis of amino acids | Tryptophan | 6.763 | 204.0898 | Glycine, serine and threonine metabolism; Tryptophan metabolism; Phenylalanine, tyrosine and tryptophan biosynthesis; Indole alkaloid biosynthesis; Glucosinolate biosynthesis; Aminoacyl-tRNA biosynthesis; Metabolic pathways; Biosynthesis of secondary metabolites; 2-Oxocarboxylic acid metabolism; Biosynthesis of amino acids |
|  | L-Saccharopine | 1.204 | 276.1316 | Lysine biosynthesis; Lysine degradation; Metabolic pathways; Biosynthesis of secondary metabolites; Biosynthesis of amino acids |
|  | L-Argininosuccinate | 1.198 | 290.122 | Arginine biosynthesis; Alanine, aspartate and glutamate metabolism; Metabolic pathways; Biosynthesis of secondary metabolites; Biosynthesis of amino acids |
|  | N-Acetyl-L-glutamate | 1.054 | 189.064 | Arginine biosynthesis; Metabolic pathways; Biosynthesis of secondary metabolites; 2-Oxocarboxylic acid metabolism; Biosynthesis of amino acids |

| **Table S8. Target gene primer list** | | |  |
| --- | --- | --- | --- |
| **Gene ID** | **Forward primer** | **Reverse primer** |  |
| DG3C06505 | CACTACTCCAAGACATGCCCGAATG | AAGCAGCACCGATCCATCACAAC |  |
| DG3C05831 | CCGAGGACTTGTAGCTTGCATGAG | GTGTGTGAGAATATCCCGCCGTAAC |  |
| DG7C00992 | GTCAGGGATGCTCATAGACCACAAC | GTCGTTCTCGTCCATCTTCGCTAC |  |
| DG3C02730 | AAAGTGCTGAGGTTGGTGGTGATC | TCCTCGTCGTCGTCGTACTTCTC |  |
| DG1C05385 | GATCCTGCTCAACTTCCAGTCGTAC | GCTTTCCTGCCGCTTCCTCATC |  |
| DG5C01300 | TGCTGCTGCTGGACGTTGTATC | AGACGGTTCGCTTGACTTCCTTTC |  |
| DG6C01655 | ACCAGACTCCAGAAGGTTCACTCC | GAGGAACGGTATCTCTGAACGCAAG |  |
| DG7C02648 | TCAACAAGATGCGGTCCAACTGAG | GTACTTACACCACGTCGAGTTCCAC |  |
| DG7C02886 | CCCAGAGCGGAGGTCCTTACTAC | GGAGAAGAAGGCGTTGAGCTTGTC |  |
| DG6C03517 | CGATGCTCAGAGATGGTGGTGTG | CTGCGAAACGGAACAAATGGAAGAC |  |
| DG7C01296 | ACTGCTGTGAAAGTGGTGCTGATC | TTCGCCGAGGATTCTGTTACAACTG |  |
| Actin | GATCTTCGCCTCGCCAGGTTATC | ATATCGCCGTGCTTCATCCATGTC |  |
|  |  |  |  |

| **Tabie S9. Chromatographic elution gradient table** | | |  |
| --- | --- | --- | --- |
| **Time (min)** | **A%** | **B%** |  |
| 0 | 98 | 2 |  |
| 1.5 | 98 | 2 |  |
| 3 | 15 | 85 |  |
| 10 | 0 | 100 |  |
| 10.1 | 98 | 2 |  |
| 11 | 98 | 2 |  |
| 12 | 98 | 2 |  |

| **Table S10. Instruments used for metabolite detection** | | | |  |
| --- | --- | --- | --- | --- |
| **Instrument type** | **Type** | **Brand** | **Origin** |  |
| Low temperature centrifuge | D3024R | Scilogex | USA |  |
| Chromatographic column | Hypesil Gold column | Thermo Fisher | USA |  |
| Chromatograph | Vanquish UHPLC | Thermo Fisher | Germany |  |
| Mass spectrometer | Q Exactive™ HF | Thermo Fisher | Germany |  |

| **Table S11. Reagents for metabolite detection** | | | | |
| --- | --- | --- | --- | --- |
| **Reagent** | **Purity** | **CAS** | **Brand** | **Origin** |
| Methanol | LC-MS Grade | 67-56-1 | Thermo Fisher | USA |
| Water | LC-MS Grade | 7732-18-5 | Merck | Germany |
| Formic acid | LC-MS Grade | 64-18-6 | Thermo Fisher | USA |
| Ammonium acetate | LC-MS Grade | 631-61-8 | Thermo Fisher | USA |
