# Supplementary material for: Integrated Transcriptomic and Metabolomics Analysis of the Root Responses of Orchardgrass to Submergence Stress
Source: Int J Mol Sci. 2023 Jan 20;24(3):2089. doi: 10.3390/ijms24032089 (PMC9916531; doi:10.3390/ijms24032089)
Supplement: Supplementary file 1 [file ijms-24-02089-s001.zip › Supplemental figures.pptx]

## Slide 1
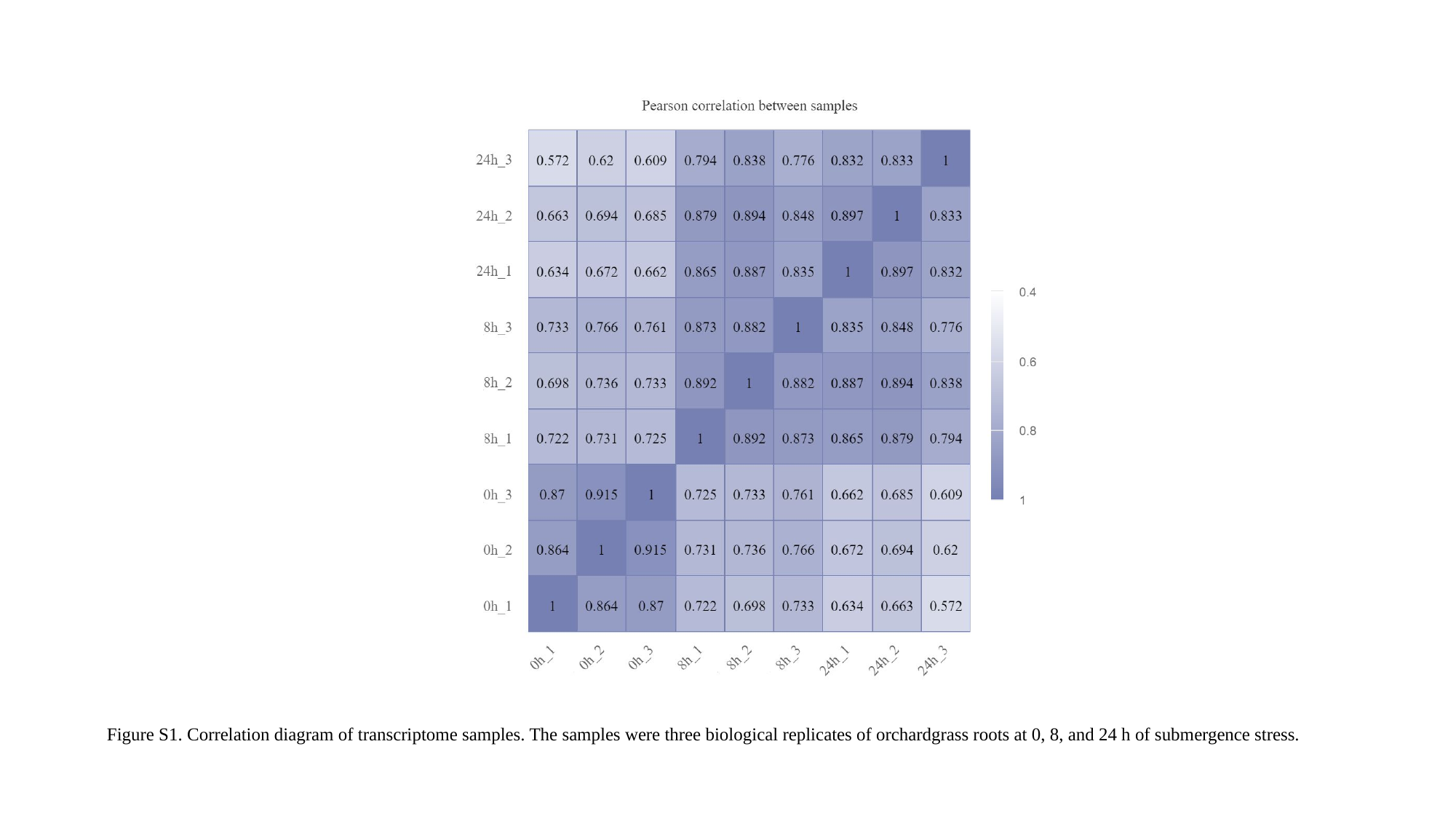

Figure S1. Correlation diagram of transcriptome samples. The samples were three biological replicates of orchardgrass roots at 0, 8, and 24 h of submergence stress.

## Slide 2
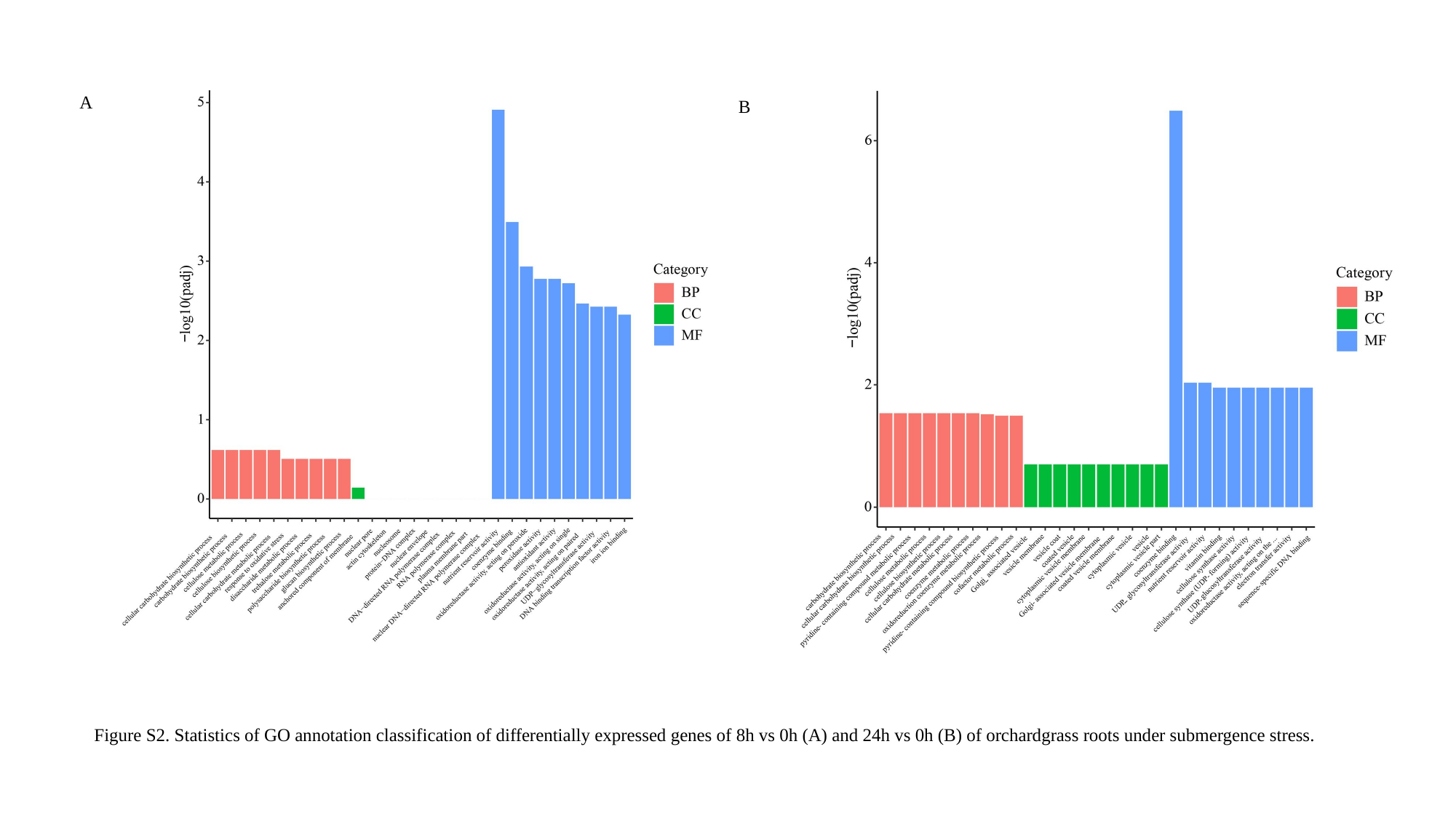

A
B
Figure S2. Statistics of GO annotation classification of differentially expressed genes of 8h vs 0h (A) and 24h vs 0h (B) of orchardgrass roots under submergence stress.

## Slide 3
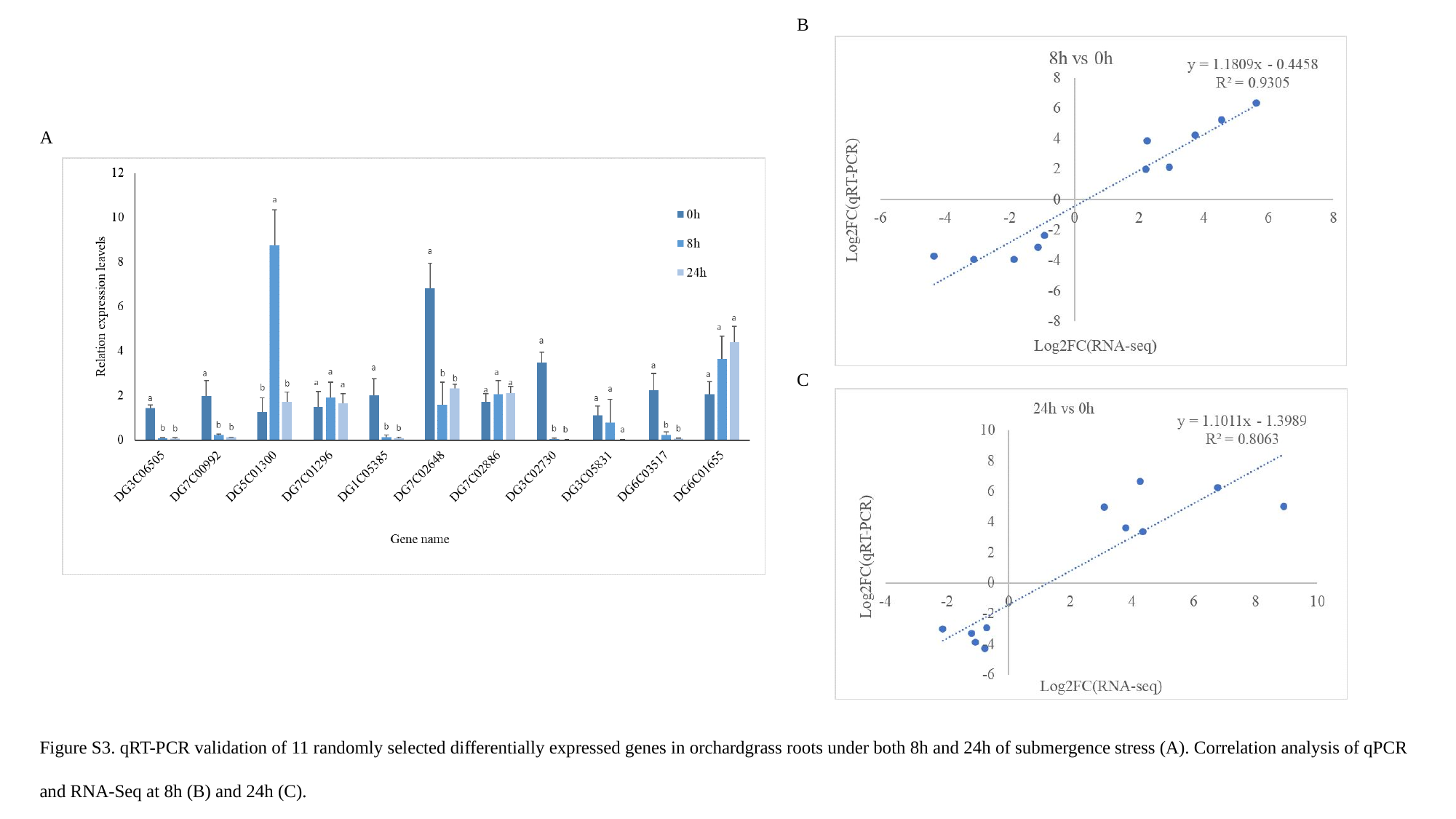

B
A
C
Figure S3. qRT-PCR validation of 11 randomly selected differentially expressed genes in orchardgrass roots under both 8h and 24h of submergence stress (A). Correlation analysis of qPCR and RNA-Seq at 8h (B) and 24h (C).

## Slide 4
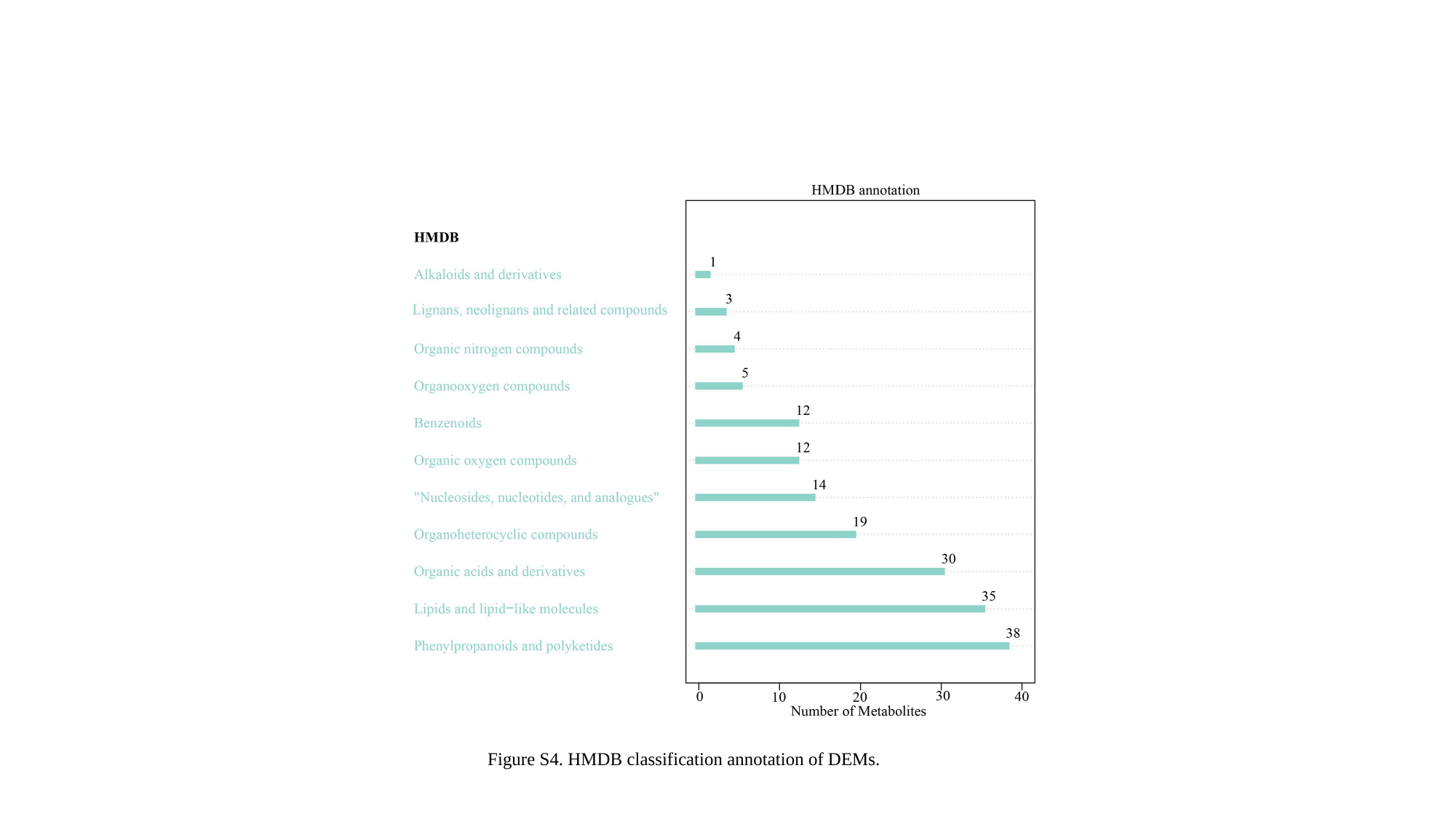

Figure S4. HMDB classification annotation of DEMs.

## Slide 5
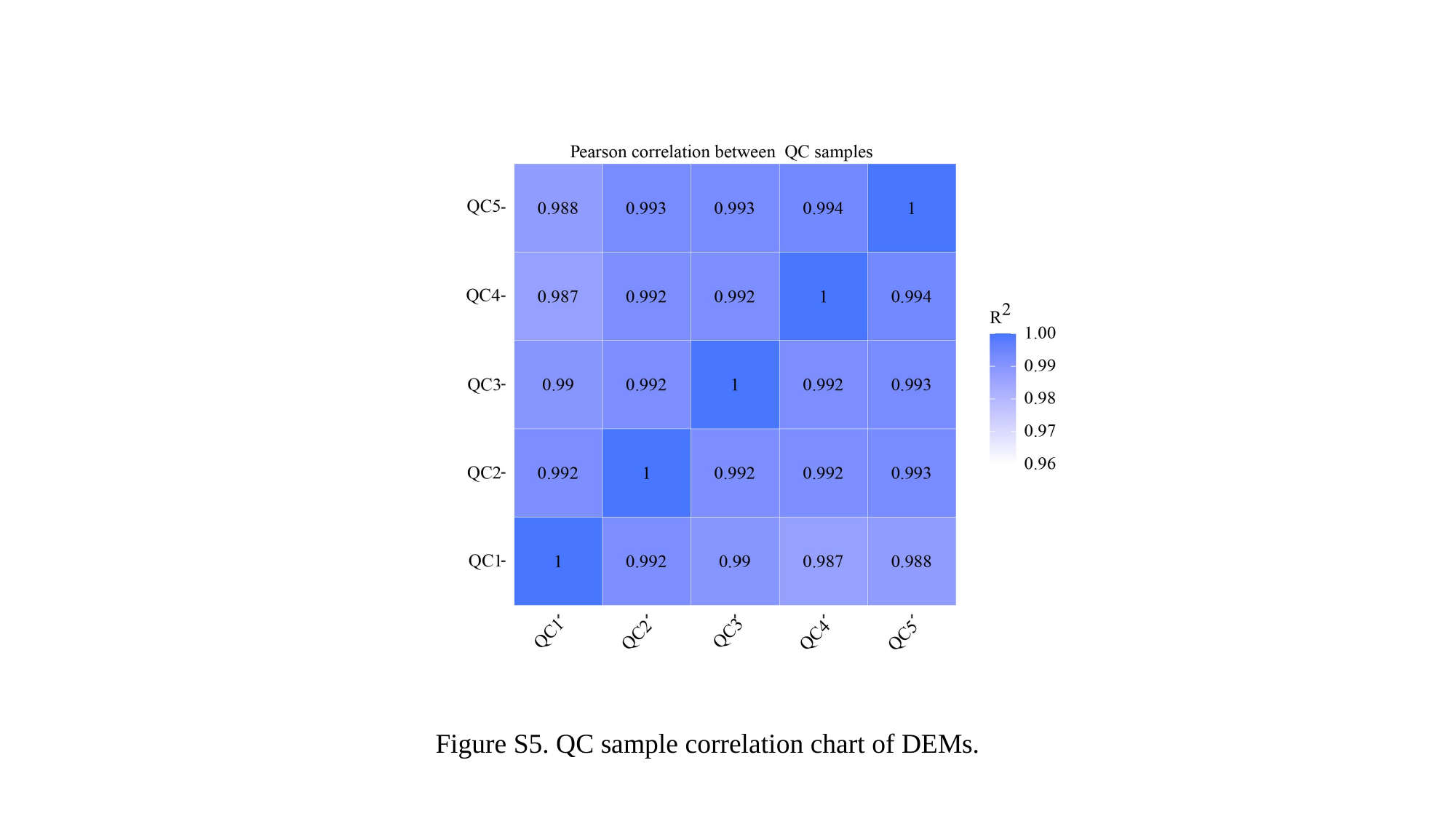

Figure S5. QC sample correlation chart of DEMs.

## Slide 6
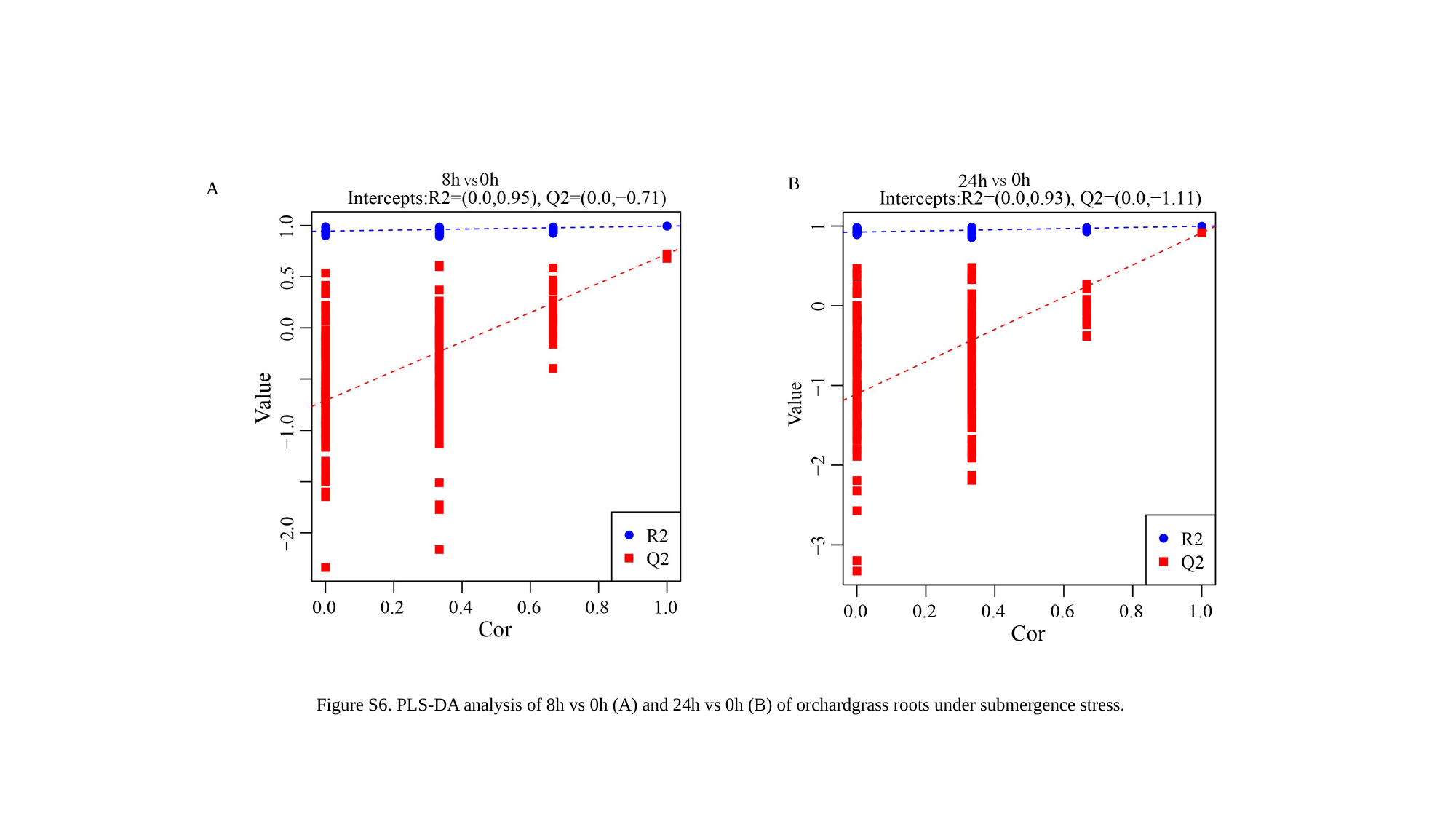

B
A
Figure S6. PLS-DA analysis of 8h vs 0h (A) and 24h vs 0h (B) of orchardgrass roots under submergence stress.

## Slide 7
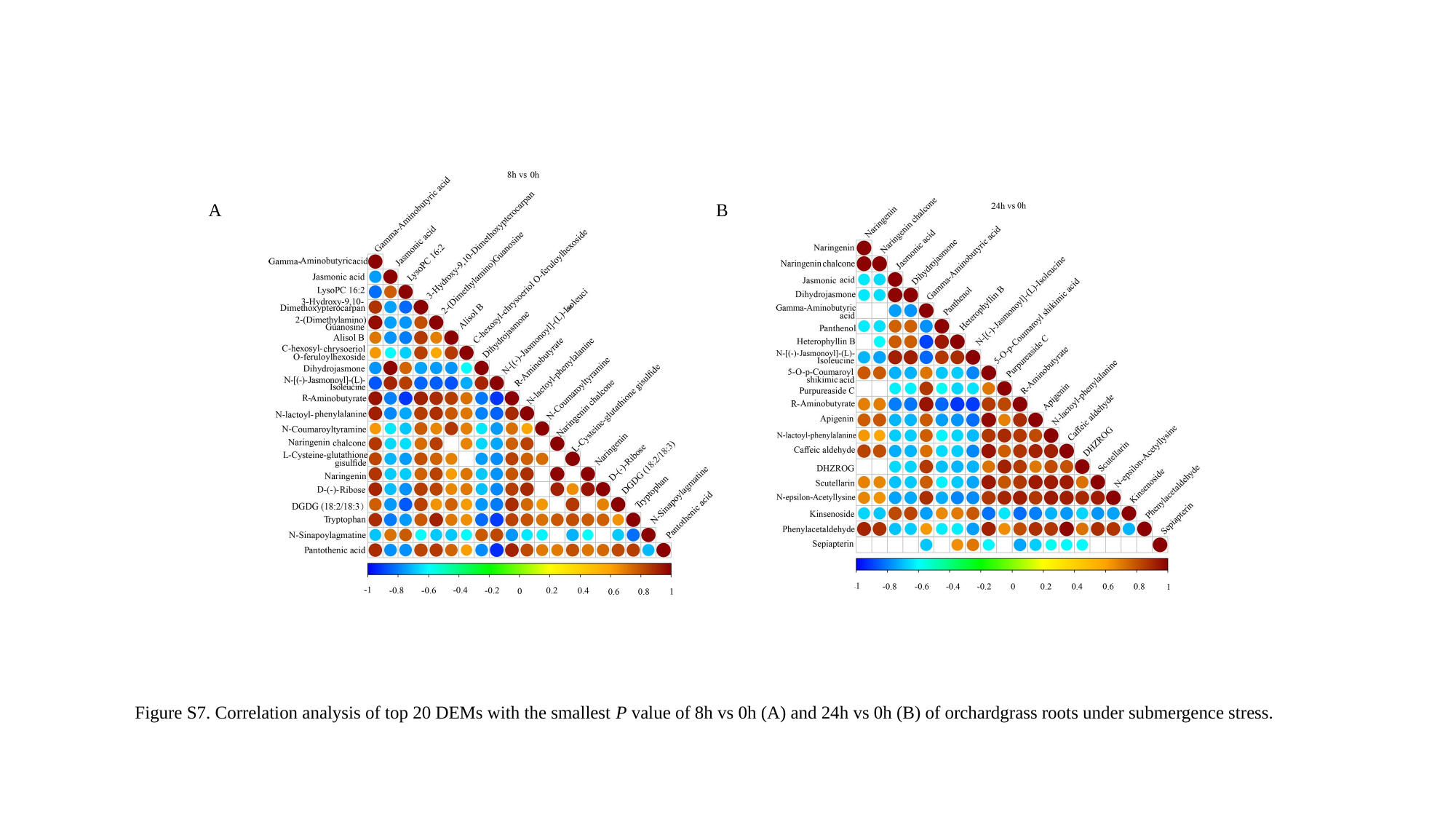

A
B
Figure S7. Correlation analysis of top 20 DEMs with the smallest P value of 8h vs 0h (A) and 24h vs 0h (B) of orchardgrass roots under submergence stress.
